# Smart Contact Lens with Dual‐Sensing Platform for Monitoring Intraocular Pressure and Matrix Metalloproteinase‐9

**DOI:** 10.1002/advs.202104738

**Published:** 2022-02-23

**Authors:** Ying Ye, Yuancai Ge, Qingwen Zhang, Meiling Yuan, Yu Cai, Kang Li, Yang Li, Ruifeng Xie, Changshun Xu, Danfeng Jiang, Jia Qu, Xiaohu Liu, Yi Wang

**Affiliations:** ^1^ School of Ophthalmology and Optometry, Eye Hospital, School of Biomedical Engineering Wenzhou Medical University Wenzhou 325027 P. R. China; ^2^ Wenzhou Institute University of Chinese Academy of Sciences Wenzhou 325001 P. R. China; ^3^ School of Opto‐Electronic Engineering Changchun University of Science and Technology Changchun 130022 P. R. China

**Keywords:** dual‐sensing platform, intraocular pressure, matrix metalloproteinase‐9, surface‐enhanced Raman scattering, smart contact lens

## Abstract

Contact lenses have become a popular health‐monitoring wearable device due to their direct contact with the eyes. By integrating biosensors into contact lenses, real‐time and noninvasive diagnoses of various diseases can be realized. However, current contact lens sensors often require complex electronics, which may obstruct the user's vision or even damage the cornea. Moreover, most of the reported contact lens sensors can only detect one analyte. Therefore, an optical‐based dual‐functional smart contact lens sensor has been introduced to monitor intraocular pressure (IOP) and detect matrix metalloproteinase‐9 (MMP‐9), both of which are key biomarkers in many eye‐related diseases such as glaucoma. Specifically, the elevated IOP is continuously monitored by applying an antiopal structure through color changes, without any complex electronics. Together with the peptide modified gold nanobowls (AuNBs) surface‐enhanced Raman scattering (SERS) substrate, the quantitative analysis of MMP‐9 at a low nanomolar range is achieved in real tear samples. The dual‐sensing functions are thus demonstrated, providing a convenient, noninvasive, and potentially multifunctional sensing platform for monitoring health and diagnostic biomarkers in human tears.

## Graphical abstract

Intraocular pressure (IOP) and matrix metalloproteinase‐9 (MMP‐9) are biomarkers closely associated with ocular diseases. A dual‐functional contact lens sensor for detections of both biomarkers, using structural colors and peptides modified surface‐enhanced Raman scattering (SERS) substrate, respectively is reported. This optical sensor, therefore, provides a potentially versatile, noninvasive sensing platform for detecting multibiomarkers of human eye diseases.

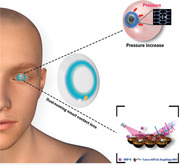

## 1 Introduction

Wearable biosensors are physiological monitoring devices for disease diagnosis that provide real‐time detection of biomarkers through noninvasive measurements.^[^ ^1^ ^]^ Its purpose is to closely monitor the physiological state of individuals without interferences or restricting the movement of users. It can be used not only to monitor the vital signs of the human body, such as pulse,^[^ ^2^ ^]^ temperature,^[^ ^3^ ^]^ etc., but also to monitor metabolites from various parts of the body, such as glucose,^[^ ^4^ ^]^ alcohol,^[^ ^5^ ^]^ drug derivatives,^[^ ^5^, ^6^ ^]^ etc. Within all parts of the human body, the eyes and relevant solutions like tears provide important physiological signals, such as intraocular pressure (IOP), corneal thickness, and various biomarkers in tears that diffuse from the blood through the tear‐blood barrier. Therefore, tears can be used as a potential substitute of blood for reducing the pain and inconvenience caused to patients during blood collection, to achieve noninvasive detection of certain diseases. For instance, commercially viable use of qualitative analysis of matrix metalloproteinase‐9 (MMP‐9) in tears for the diagnosis of dry eye,^[^ ^7^ ^]^ and diabetes could be diagnosed by quantitative detection of glucose in tears as well.^[^ ^8^ ^]^ Moreover, compared with the blood, the compositions in tears are simpler and have less interference from other substances during the detection progress. Thus, designing a wearable biosensor for the eyes and tear is of great significance for the noninvasive and real‐time monitoring of health conditions.

Contact lenses are mainly used for vision correction and cosmetic purposes. Since they are worn on the eyes and constantly in contact with tears, they can provide a unique wearable platform for diagnosing diseases. As early as 1974, Gillman and Green pioneered the first noninvasive IOP monitoring contact lens sensor based on the concept that changes in IOP lead to changes in the radius of curvature of the eye.^[^ ^9^ ^]^ Subsequently, more and more contact lens sensors have been proposed and developed aiming to detect the physical and chemical biomarkers present in the eyes.^[^ ^10^ ^]^ In 2016, the first contact lens sensor for 24 h IOP monitoring was officially approved by the U.S. Food and Drug Administration for marketing in the United States.^[^ ^11^ ^]^ Most recently, Ku et al. developed a contact lens sensor integrated with electronic components to monitor cortisol concentrations in human tears in real‐time, aiming to quantitatively analyze the stress levels in humans.^[^ ^12^ ^]^ Therefore, the contact lens is a promising wearable platform for real‐time monitoring of biomarkers to provide useful diagnosis and treatment information for diseases. However, the current development of contact lens sensors also facing some problems, such as the damage to the eyes caused by electronic components, the requirements for power supply, and so on.^[^ ^13^ ^]^ In addition, the function of the contact lens sensor is relatively simple, which in general only enables single target detection.

Herein, we report an optical‐based dual‐function contact lens sensor for IOP monitoring and MMP‐9 detection. Increased IOP is the main risk factor for glaucoma, which is the second leading cause of irreversible blindness worldwide.^[^ ^14^ ^]^ Currently, the measurement of IOP is mainly achieved through a tonometer, which often needs to be performed in the clinic. However, the diagnostic accuracy of IOP values obtained by continuous monitoring for 24 h is nearly 50% higher than that obtained by measurement in the office.^[^ ^15^ ^]^ Therefore, continuous IOP monitoring outside the clinics is very much preferred. MMP‐9 is a nonspecific inflammation biomarker with the capacity to cleavage both elastin and partially hydrolyzed collagen, which plays an important role in wound healing and inflammation.^[^ ^16^ ^]^ It is also a biomarker of glaucoma, in which the MMP‐9 is overexpressed in the tears of patients with early glaucoma.^[^ ^17^ ^]^ Moreover, elevated IOP has been found to induce activation of MMP‐9, which ultimately leads to retinal dysfunction.^[^ ^18^ ^]^ Currently, the RPS InflammaDry Detector (Rps Diagnostics, Inc. USA) provides a fast way to detect MMP‐9 levels in tears based on immunoassays.^[16a]^ However, this method can only be used for the qualitative analysis of MMP‐9 when the concentration is higher than 40 ng mL^−1^, thus determining the presence of the related disease. In order to assess the severity of the disease, such as glaucoma, simultaneous and quantitative analysis of both IOP and MMP‐9 is required.

**Figure 1:**
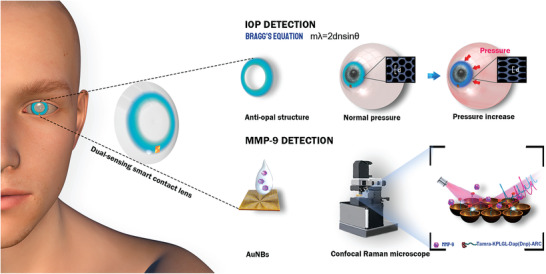
Schematic illustration of the dual‐functional contact lens sensor, which is composed of the antiopal structure for IOP monitoring and the peptide‐functionalized AuNBs SERS substrate for MMP‐9 detection.

The optical‐based dual‐function contact lens sensor developed in this contribution aims to achieve the continuous monitoring of IOP via structural color, and the quantitative analysis of MMP‐9 via surface‐enhanced Raman scattering (SERS), respectively. The structural color is produced by the selective diffraction of light from a unique 3D periodic structure and can be changed by adjusting the refractive index or lattice spacing of the periodic structure. The contact lens employing structural colors overcomes the potential harm to the eyes and becomes a suitable IOP monitoring platform. Meanwhile, the SERS technology is a fast and sensitive spectroscopic analysis tool, which can provide label‐free identification and quantitative detection of different molecules for a wide range of applications in the analytical, biological, and chemical fields.^[^ ^19^ ^]^ The layout and the overall structure of the dual‐functional “Structural color‐SERS” smart contact lens, including the ringlike antiopal structure and the gold SERS substrate are illustrated in **Figure** **1**. First of all, both the antiopal structure and gold substrate are located on the outer ring of the contact lens to avoid blocking vision. For the IOP monitoring, IOP increasement in the eyeball leads to the corneal curvature radius of the eyeball increase, stretching the contact lens as well as the antiopal structure and resulting in the color changes. For the MMP‐9 detection, a rationally designed peptide substrate with a fluorescent Raman tag is employed to ensure the specificity based on SERS, where the signals are enhanced by a monolayer consisting of homogeneous gold nanobowls (AuNBs). In the presence of MMP‐9, the peptides functionalized on AuNBs are cleaved together with the Raman tag, 5‐carboxytetramethyl rhodamine (Tamra), followed by a reduced Raman signal. The dual‐functional sensing platform of smart contact lens is therefore assembled and subsequently applied on porcine eyes for simultaneous detections of IOP and MMP‐9.

## 2 Results and Discussion

### 2.1 Characterization of Structural Color Contact Lens

**Figure 2:**
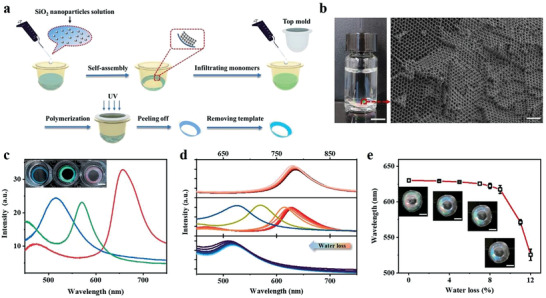
a) The schematic diagram of the preparation process of structural color contact lens, including nanoparticles self‐assembly, monomer addition, polymerization, and template removing. b) The photograph of a structural color contact lens (left) and the scanning electron microscopy (SEM) image of antiopal structure on contact lens after etching SiO_2_ (right). Scale bars: 5 mm (left) and 1 µm (right). c) Reflection spectra of structural color contact lenses with blue, green, and red colors. Inset: their corresponding photographs. Scale bar: 5 mm. d) The blueshift of the reflection spectra of different colored contact lenses as the water content reduced. From top to bottom: red lens, green lens, and blue lens. e) Blueshift of the reflection peaks as increasing the percentage of water loss. All data were presented as mean ± standard deviation (s.d.) (*n* > 3). Inset photos: apparent color changes at each water loss stage. Scale bar: 5 mm.

The contact lenses utilized in this work were prepared via an ordinary route. Briefly, the monomer solution was added into the contact lens molds, followed by polymerization under ultraviolet (UV) light. The properties of the as‐prepared contact lenses were close to commercial contact lenses in terms of water content, oxygen permeability, and light transmittance (Table S1, Supporting Information). The structural color contact lenses were then further developed. The general process of preparing structural color contact lenses included nanoparticles self‐assembly, monomer adding, polymerization, and template removing as presented in **Figure** **2**a, where the color was generated by the antiopal structure of the polymer itself. A detailed description of the whole process could be found in the Experimental Section. Three kinds of photonic crystal templates with different colors were self‐assembled by using monodisperse silica (SiO_2_) nanoparticles with three different sizes (180, 240, 300 nm) (Figure S1, Supporting Information). The structural color contact lenses were subsequently obtained after completely removing the template using the hydrofluoric acid (HF) etching technique, with compact and porous periodic anti‐opal structures (Figure 2b; and Figure S2, Supporting Information). As the average size of monodisperse SiO_2_ nanoparticles decreased, the reflectance spectra of the contact lenses showed a significant blue shift, and the corresponding colors appeared to be red, green, and blue, respectively (Figure 2c). All three structural colored lenses were tested for the color changes as gradually losing water content recorded by the UV–visible spectrometer. The spectra obtained from different lenses had blueshifts when the water content of the lenses reduced, in which the green‐colored lens showed a more pronounced change (Figure 2d). However, at the low level of water loss (< 5%), all three lenses had limited spectral shifts and the colors remained (Figure S3, Supporting Information). Whereas the green‐colored lens started to show an obvious change at a relatively high level of water loss (> 5%) than the other two (Figure 2e; and Figure S3, Supporting Information). The inset photos of Figure 2e showed the real colors change of the green‐colored lens at different levels of water loss, especially when it exceeded 9% of water loss the spectral shift accelerated and moved out of the visible range quickly. Therefore, the green lens was accordingly selected for further investigations of IOP monitoring.

### 2.2 Ex Vivo IOP Monitoring on the Porcine Eye

**Figure 3:**
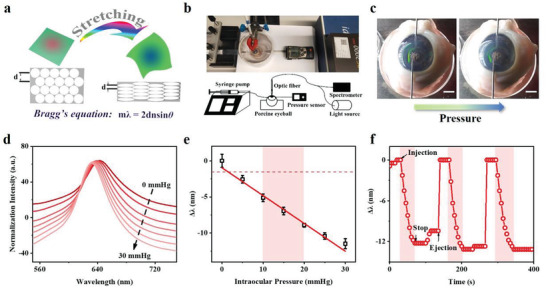
a) Schematic illustration of the working principle for the IOP monitoring by structural color contact lenses. b) The photograph and scheme (bottom) of the optical device setup for measuring IOP on a porcine eye using a structural color contact lens sensor. c) The photograph obtained by the structural color contact lens sensor worn on the porcine eye during the real‐time IOP monitoring (increasing the pressure). Scale bar: 5 mm. d) The blue shift in the reflectance spectra of the contact lens on a porcine eye when applying IOP up to 30 mmHg. e) The corresponding linear calibration curve (*R* ^2^ = 0.978) of the blue shift of the reflection peaks versus IOP increasing in d). The red broken line referred to 3 times of s.d. for the limit of detection (LOD) estimation. The red shaded region referred to the physiological status. All data were presented as mean ± s.d. (*n* > 3). f) The cyclic real‐time tests of the contact lens for IOP monitoring. The red shaded region referred to IOP increment by injection of fluids.

Due to the similar sizes between the enucleated porcine eyes and the human eyes, it was suitable for the structural color contact lenses to monitor IOP ex vivo on porcine eyes.^[^ ^20^ ^]^ The average Young's modulus of the porcine cornea was reported to be larger than that of the human cornea, which caused the deformation of the porcine cornea would be smaller than the human cornea under the same pressure change.^[10a]^ Therefore, monitoring IOP on porcine eyes enabled the sensor platform to meet the requirements for future clinical applications in humans. The working principle of the structural color contact lens was illustrated in **Figure** **3**a. When the IOP increased, the corneal curvature radius of the eyeball increased, followed by the deformation of the contact lens as well as the stretching of the antiopal structure, which eventually led to a blue shift of the reflectance spectra. The blue shift of the refractive wavelength was expected based on the following Bragg's equation

(1) $$\lambda \;=\;\frac{2\;d\;n_{\mathrm{ave}}\;\sin\theta }{m}$$

where *λ* is the wavelength, *d* is the lattice spacing, *n*_ave_ is the average refractive index of the poly(2‐hydroxyethyl methacrylate) (PHEMA) hydrogel, *θ* is the oblique angle between the incident light and the diffracting crystal plane, and *m* is the diffraction order (i.e.*, λ* decreases as *d* decreases when stretching).

The experimental device for conducting IOP monitoring consisted of a syringe pump, a pressure sensor, and a spectrometer system (Figure 3b). The constant pressure input was achieved by injecting water into the eyeball where the internal pressure was continuously monitored by the pressure sensor. In addition, a cover was put over the porcine eye with water drops around it to maintain the humidity in order to avoid the interference of the IOP monitoring caused by the water loss. The color changed from green to greenish‐blue in the contact lens with the increment of the pressure in the porcine eye (Figure 3c). A series of pressures in the eyeball was then monitored and the corresponding spectra were obtained. As previously expected, a gradual blueshift was observed which was consistent with the expectation derived from Bragg's equation (Figure 3d). The blue shift of spectral peaks was slowing down after IOP exceeding 30 mmHg and tended to be flat up to 50 mmHg (Figure S4, Supporting Information). This might be ascribed to the volume limitation of the porcine eyeball, restraining the radius of curvature to further increase. Nevertheless, there was a good linear detection range of IOP from 0 to 30 mmHg (fitted line with *R* ^2^ = 0.978), covering the physiological IOP range that was usually located at 10–20 mmHg (Figure 3e). The real‐time monitoring of IOP was then conducted in cycles, simulated by the fluid injection into or withdrawal from the eyeball. A similar pattern could be derived for each cycle, in which increment of IOP (injection) caused the gradual blue shift of the spectral peaks and paused at the maximum IOP followed by the fast recovery step (ejection) which enabled the next round (Figure 3f; and Figure S5a, Supporting Information). Furthermore, the monitoring of IOP could perform equally well in the bovine serum albumin (BSA) concentrated buffer (10 mg mL^−1^) without any observable abnormals (Figure S5b, Supporting Information). The structural color contact lenses were successfully developed, optimized, and applied to ex vivo IOP real‐time monitoring within the physiological meaningful range.

### 2.3 Characterization of AuNBs SERS Substrate

**Figure 4:**
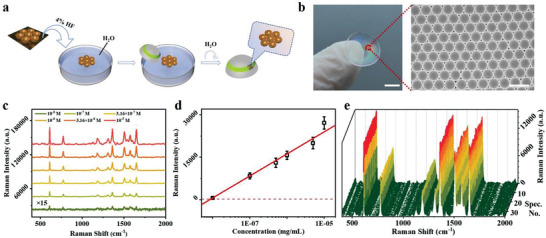
a) The schematic process of transferring the AuNBs substrate from the SiO_2_/Si wafer to the contact lens. b) The photograph of AuNBs substrate on the structural color contact lens (left) and the SEM image of AuNBs substrate (right). Scale bar: 5 mm (left) and 1 µm (right). c) Raman spectra with different concentrations of R6G on the AuNBs SERS substrate (10^−5^ m, 3.16×10^−5^ m, 10^−6^ m, 3.16×10^−6^ m, 10^−7^ m, and 10^−8^ m, from red to green, respectively). d) The linear calibration curve (*R* ^2^ = 0.991) of Raman intensities at 613 cm^−1^ with different concentrations of R6G in c), the red broken line referred to 3 times of s.d. for LOD estimation. All data were presented as mean ± s.d. (*n* > 3). e) Raman signal intensities of R6G (10^−7^ m) at 613 cm^−1^ randomly recorded from the AuNBs SERS substrate (*n* = 30).

The AuNBs SERS substrate was a homogeneous nanobowl monolayer structure, prepared by the metal film‐coated nanospheres structure.^[19a, 21]^ The preparation process was shown in Figure S6 (Supporting Information). Briefly, the polystyrene (PS) opal structure was first formed on the water surface and then transferred to the silicon (Si) wafer (Figure S7a, Supporting Information). A 150 nm thick Au layer was sputtered on the 2D‐PS opal structure to obtain the Au‐coated opal template (Figure S7b, Supporting Information). The Au‐coated PS template was transferred upside down to the SiO_2_/Si wafer via the air–water interface. The purpose of switching to SiO_2_/Si wafer was that HF could etch off the SiO_2_ on the upper layer of the wafer and reduce the force between AuNBs and the wafer, thus facilitating the transfer of AuNBs later. The AuNBs substrate was obtained eventually after removing PS nanospheres with chloroform (Figure S6, Supporting Information). Furthermore, it had to be transferred again onto the structural colored contact lenses in a way shown in **Figure** **4**a, facilitated by diluted HF. Due to the homogeneous hydrophilicity, the monolayer of AuNBs substrate could be tightly bound to the contact lens (Video S1, Supporting Information). The final product of AuNBs‐loaded contact lens with the well‐maintained nanostructure was shown in Figure 4b.

To investigate the SERS performance of the AuNBs substrate, the rhodamine 6G (R6G) molecule was used. The AuNBs substrate was straightforwardly evaluated by analyzing the Raman spectra of R6G in the concentration range of 10^−8^ m to 10^−5^ m. Specifically, a series concentration of R6G (10^−5^ m, 3.16×10^−5^ m, 10^−6^ m, 3.16×10^−6^ m, 10^−7^ m, 10^−8^ m) was investigated, providing a clear dose‐dependent manner with the Raman intensities (Figure 4c). The linear calibration curve (R^2^ = 0.991) was derived according to the concentrations and the R6G Raman intensities at 613 cm^−1^ (Figure 4d). In order to estimate the LOD of R6G on AuNBs substrate, the value of the signal‐to‐noise ratio was set to be 3, with the s.d. roughly at 111.1 (based on 30 measurements of 10^−8^ m R6G). Hence, the LOD of R6G by SERS was determined to be 9.17×10^−9^ m. And the enhancement factor of AuNBs substrate was calculated to be 3.09×10^5^, providing the *C*_SERS_ and *C*_bare_ were 10^−8^ m and 10^−2^ m, and the acquired *I*_SERS_ and *I*_bare_ were about 627.1 and 2026.7 counts, respectively (Figure S8, Supporting Information). The homogeneity of the AuNBs SERS substrate was also evaluated by measuring the 10^−7^ m R6G at 30 randomly selected points on the substrate (Figure 4e). The Raman signals exhibited relative uniformity at arbitrary positions and the calculated relative s.d. is 5.74% (Figure S9a, Supporting Information). The Raman mapping of R6G at the concentration of 10^−7^ m was further collected on AuNBs substrate over an area of µm^2^ at the specific peak of 613 cm^−1^, which showed a nearly identical color gamut (Figure S9b, Supporting Information). In summary, the AuNBs substrate had been proven to be a good SERS platform for quantitative sensing.

### 2.4 MMP‐9 Detection on Contact Lens Loaded with Peptide Modified AuNBs

**Figure 5:**
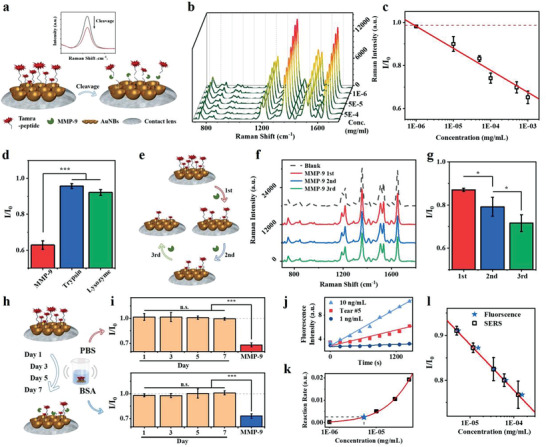
a) The schematic diagram of the Tamra‐pep cleavage by MMP‐9 on the AuNBs SERS substrate attached to contact lenses. b) The Raman spectra of Tamra‐pep on AuNBs after treating with different concentrations of MMP‐9 (1, 10, 50, 100, 500 ng mL^−1^, 1 µg mL^−1^). c) The linear calibration curve (*R* ^2^ = 0.979) of the Raman intensities difference ratios at 1648 cm^−1^ for MMP‐9 detections, the red broken line referred to 3 times of s.d. derived from signals of blank samples. All data were presented as mean ± s.d. (*n* > 3). d) SERS responses of Tamra‐pep modified AuNBs substrate to MMP‐9 (1 µg mL^−1^, red), trypsin (1 µg mL^−1^, green), and lysozyme (1 µg mL^−1^, blue). All data were presented as mean ± s.d. (*n* = 3). Statistical analyses were performed by Student's *t*‐test, *p* < 0.001. e) The schematic illustration of the repetitive test for MMP‐9 detections on the same smart contact lenses. f) The Raman spectra of Tamra‐pep on AuNBs after each round of incubation with MMP‐9 (10 ng mL^−1^) for 3 times. g) SERS responses of Tamra‐pep modified AuNBs substrate after the first (red), the second (blue), and the third (green) round of MMP‐9 treatment. All data were presented as mean ± s.d. (*n* > 3). Statistical analyses were performed by Student's *t*‐test, *p* < 0.05. h) The schematic illustration of the stability test for MMP‐9 detections on the same smart contact lenses over 7 days. i) SERS intensity ratios at 1648 cm^−1^ of Tamra‐pep modified AuNBs substrate on Day 1 to Day 7 and after cleavage of MMP‐9 immersed in phosphate buffered saline (PBS) (red, top) and PBS with 9 mg mL^−1^ BSA (blue, bottom). All data were presented as mean ± s.d. (*n* > 3). Statistical analyses were performed by Student's *t*‐test, n.s., not significant; *p* < 0.001. j) The fluorescence kinetics curves of 10 ng mL^−1^ MMP‐9 (light blue triangles), Tear #5 (red squares), and 1 ng mL^−1^ MMP‐9 (blue dots). k) The MMP‐9 level of Tear #5 (blue star) marked in the calibration curve of the fluorescent assay. l) The measured concentrations of MMP‐9 in the spiked Tear #5 samples (black squares) according to the SERS calibration curve (red line) aligned with the calculated concentrations obtained by the fluorescent assay (blue stars). All data were presented as mean ± s.d. (*n* > 3).

MMP‐9 was a well‐known inflammation biomarker with enzymatic cleavage properties, which could specifically cleave a peptide substrate containing amino acids glycine (Gly) and leucine (Leu). Thus, a peptide‐based quantitative assay for MMP‐9 was developed by using AuNBs SERS substrate on the smart contact lens. Prior to any evaluations, the enzymatic activity of MMP‐9 was first confirmed by incubating with a fluorescent‐quenched peptide substrate. The sequence of this peptide was Mca‐Lys‐Pro‐Leu‐Gly‐Leu‐Dap(Dnp)‐Ala‐Arg, where Mca referred to the fluorescent methoxy‐coumarin‐acetic‐acid and Dnp referred to the efficient quencher, 2,4‐dinitrophenyl. Besides, Dap is the nonproteinogenic amino acid, L‐2,3‐diaminopropionic acid, used to link Dnp to the peptide chain.^[^ ^22^ ^]^ Under the fluorescent plater reader, the intensities of unquenched Mca could be readily recorded as the MMP‐9 chopped the peptide substrate and separated Mca and Dnp (Figure S10a, Supporting Information). The initial enzymatic reaction rates depended on the various concentrations of MMP‐9 in a positive correlated way (Figure S10b, Supporting Information). The same detection principle of MMP‐9 was applied to AuNBs substrate, with another adopted peptide. The sequence was designed as Tamra‐Lys‐Pro‐Leu‐Gly‐Leu‐Dpa‐Ala‐Arg‐Cys (Tamra‐pep), where Tamra referred to 5‐carboxytetramethyl rhodamine. Compared with the previous fluorescent peptide, Tamra was used to replace Mca to serve as the Raman tag, while a terminal cysteine was also added for the immobilization of peptides onto the gold surface via a thiol—Au bond. A schematic diagram for MMP‐9 detection on the smart contact lens was shown in **Figure** **5**a. The specific Tamra‐pep was pre‐immobilized on the AuNBs, which were specifically recognized and cleaved by the MMP‐9 at the Gly‐Leu site, resulting in the release of Tamra terminal and decrease of the Raman intensity. The Raman spectra of the Tamra‐pep on AuNBs were measured upon the treatment with different concentrations of MMP‐9 (Figure 5b). The characteristic peaks of Tamra at 1648 cm^−1^ (corresponding to aromatic C—C stretching) and 1356 cm^−1^ (corresponding to the C—O stretching) dropped significantly as the concentration of MMP‐9 increased, proving that the target protein could cleave the peptide substrates to free the Tamra from the SERS substrate. The corresponding linear calibration curve (*R* ^2^ = 0.979) was then plotted derived from the acquired spectra, where the ratios of Raman intensities at 1648 cm^−1^ (I) and the original intensity (I_0_) were utilized (Figure 5c). The LOD of MMP‐9 was estimated to be 0.90 ng mL^−1^ according to the fitted line with 3 times of s.d. (*σ*_contact lens_ = 0.986) derived from signals of blank samples. As a matter of fact, the Tamra‐pep on AuNBs cleaved by a series of concentrations of MMP‐9 was first investigated on the SiO_2_/Si wafer (Figure S11a,b), in order to check the viability. Interestingly, the SERS sensor on SiO_2_/Si wafer showed a wider detection linear range (0.1 ng mL^−1^ to 1 µg mL^−1^) than that on the contact lens (1 ng mL^−1^ to 1 µg mL^−1^), as well as a lower LOD of 0.29 ng mL^−1^ (Figure S11c, Supporting Information). This might be ascribed to the trapping and/or nonspecific adsorption of MMP‐9 on the contact lens hydrogels thus reducing the amount of enzyme participating in the cleavage reaction.^[^ ^23^ ^]^

The specificity was investigated as well by comparing the Raman intensity of MMP‐9 with other substances (all at 1 µg mL^−1^) including trypsin and lysozyme which was a potential contaminator in tears. The Raman spectra corresponding to these three different enzymes treated peptides were collected on the contact lenses with a blank control (Figure S12a, Supporting Information). The ratios of intensity differences of each enzymatic protein were calculated and compared, showing that MMP‐9 caused a much more pronounced difference than the other two, despite that trypsin is the well‐known universal enzyme (Figure 5d). The results indicated that the SERS sensor has a specific response to MMP‐9. The specificity was also evidenced by the fluorescent assay, in which the fluorescence of the quenched peptide was recovered rapidly upon the addition of MMP‐9 and reached the equilibrium at around 20 min, but no response was observed for trypsin nor lysozyme (Figure S12b, Supporting Information).

Moreover, a repetitive test was also conducted to investigate the reusability of contact lenses for the MMP‐9 detections, as some of the peptide substrates remained intact on the AuNBs after the first round of cleavage at the presence of a relatively low level of MMP‐9 (Figure 5e). It was found that the SERS signals reduced stepwise after each round of incubation with 10 ng mL^−1^ of MMP‐9 for 2 h for 3 times, suggesting MMP‐9 repetitively cleaved the remaining Tamra‐pep on the contact lens (Figure 5f). The ratios were further calculated to deduce the concentrations of MMP‐9, resulting in an average concentration of 8.7 ng mL^−1^, though the variations seemed to increase after the first round (Figure 5g). Considering the relatively low‐cost and short usage time of a contact lens, it would be enough to have 3 times of MMP‐9 testing capability for the smart contact lens.

The stability assay was carried out by immersion of the smart contact lens in the PBS or BSA buffers with extensive shaking for 7 days (Figure 5h). The SERS intensity ratios at 1648 cm^−1^ were examined continuously over the 1‐week immersion, showing small fluctuations as compared with the initial intensities (Day 1 to Day 7 columns, Figure 5i). After the final cleavages at the presence of MMP‐9 (500 ng mL^−1^) for the contact lenses in PBS, the SERS signals ratios dropped significantly to a level around 0.7, with an estimated MMP‐9 of 439 ng mL^−1^ (red, top, Figure 5i). While in the BSA‐rich buffer (with 9 mg mL^−1^ BSA), the contact lenses showed lower responses to MMP‐9 by 7.5% (blue, bottom, Figure 5i), indicating the existence of biofouling effect.

To further explore the effectiveness of our SERS contact lens sensor in complex biological body fluids, it was applied to detect MMP‐9 (500 ng mL^−1^) in the presence of highly concentrated BSA buffer (9 mg mL^−1^) as well as the real tear samples. The results suggested that there was a negative effect (≈13% reduced response as compared to controls, 500 ng mL^−1^ MMP‐9) on the cleavage reaction in such high protein‐rich fluids (Figure S13, Supporting Information). This could be the interference of the nonspecific interaction of BSA with the surface thus reducing the reaction response. For the detections in real samples, we measured the MMP‐9 levels in five different tear samples via the SERS contact lens (Figure S14a–e, Supporting Information) and the fluorescent assay (Figure S14f, Supporting Information) separately, in which the results obtained by the fluorescent assay were assumed as the references. The concentrations of MMP‐9 in the five samples were calculated to be 6.33 (5.63), 4.66 (5.05), 4.83 (5.67), 5.09 (5.90), and 5.00 (5.43) ng mL^−1^ according to previous calibration curves, respectively, which were comparable with that measured by fluorescent assay (in parentheses) with the variations from 7.7% to 14.8% (Table S2, Supporting Information). The results illustrated the feasibility of our SERS contact lens sensor for the detection of MMP‐9 in real samples. In addition, tears of patients with dry eyes were mimicked by spiking one of the healthy human tears (Tear #5 in Table S2 (Supporting Information), initial concentration as 5.43 ng mL^−1^ obtained by the fluorescent assay according to Figure 5j,k) with different concentrations of MMP‐9 (10, 30, 60, 100 ng mL^−1^). The corresponding Raman spectra of the subsequent samples were measured on the smart contact lens (Figure S15, Supporting Information), and the calibration curve was obtained based on the ratio of intensities at 1648 cm^−1^ between samples and negative controls (Figure 5l). Unsurprisingly, the results derived from the SERS method were similar to the reference concentrations, respectively (black squares and blue stars, Figure 5l), suggesting a relatively high accuracy for the quantitative analysis of MMP‐9.

### 2.5 Dual‐Sensing of IOP and MMP‐9 on Contact Lens

**Figure 6:**
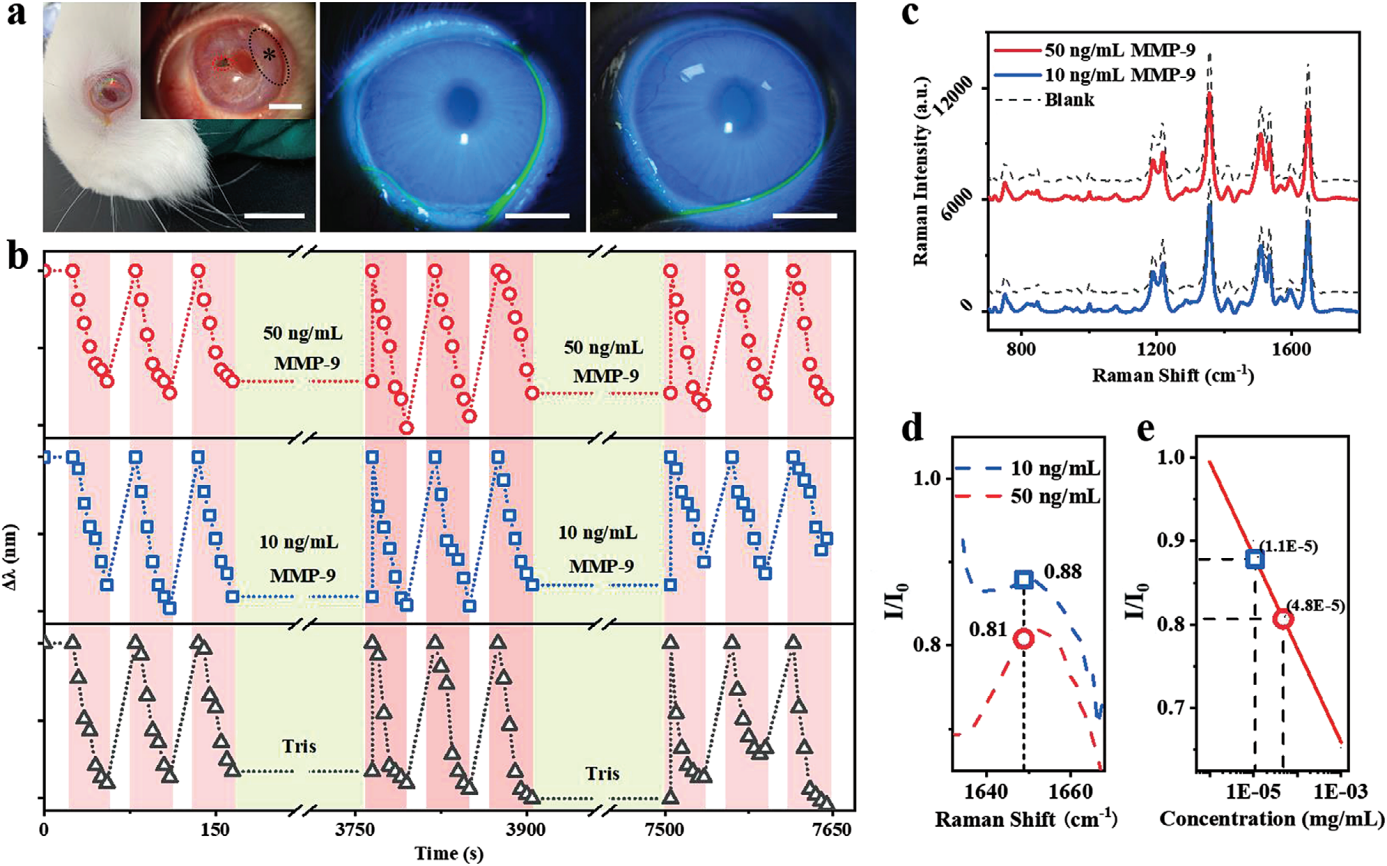
a) Photographs of the tested rabbit wearing the smart contact lens (inset: zoomed photo showing the AuNBs substrate in the red broken circle and part of structural colors in black circle) on the left. Scale bars: 20 and 5 mm (inset). Fluorescent images taken under slit lamp microscopy after the rabbits wearing the commercial contact lenses (middle) and the smart contact lenses (right). Scale bar: 5 mm. b) Real‐time monitoring of cyclic IOP increments by peak shifts of dual‐functional smart contact lens with simultaneous MMP‐9 incubations. Top: 50 ng mL^−1^ (red circles), middle: 10 ng mL^−1^ (blue squares), bottom: aminomethane hydrochloride (Tris‐HCl) buffer (black triangles). c) The Raman spectra after real‐time monitoring of IOP and 2 h incubation with 50 ng mL^−1^ (red) and 10 ng mL^−1^ (blue) with negative controls (black broken spectra). d) The ratios of Raman intensities difference between 50 and 10 ng mL^−1^ MMP‐9 incubated samples and their corresponding negative controls, respectively. e) The measured concentrations according to the calibration curve in Figure. 5c.

Before starting the dual detection, the potential toxicity of as‐assembled smart contact lenses was studied. The proliferative activity of the cells was quantified by using the Cell Counting Kit‐8 (CCK‐8), showing that both of the optical density values (O.D.) derived from cells cultured on tissue culture plates and contact lens sensors increased significantly from Day 1 to Day 3 in a similar way (Figure S16a, Supporting Information), suggesting the dual functional contact lenses were cytocompatible. Moreover, the biocompatibility was further tested on the rabbits’ eyes wearing the lenses for 8, 16, and 24 h. The representative fluorescent images showed no observable damage of the cornea and comparable results with commercial contact lenses (**Figure** **6**a; and Figure S16b, Supporting Information), confirming the good biocompatibility of our contact lens at the animal level.

In order to demonstrate that the contact lens sensor could realize the dual functions of IOP monitoring and MMP‐9 detection in the eyes, an in vitro test was carried out. For the IOP monitoring, the range was set to be 0–30 mmHg and the pressure increasing rate was controlled at 1 mmHg s^−1^, as did previously. For the MMP‐9 detection, due to the physiological concentration range of MMP‐9 in normal tears being 3–40 ng mL^−1^, two concentrations (10–50 ng mL^−1^) were selected for testing to simulate the normal and pathological state of eyes. During the measurements, the smart contact lens was first used to monitor 3 cycles of IOP changes, followed by an hour of incubation immersed in buffer or MMP‐9 solutions. The whole process was repeated and a final third IOP monitoring was done before the SERS test for MMP‐9 detection.

As shown in Figure 6a, the real‐time monitoring of IOP inside the eye was indicated by red stripes, which were separated by wider green strips for the incubation periods. The results showed clearly that the peaks blueshifted roughly to 12 nm as the IOP increased to 30 mmHg in every cycle which was consistent with previous findings, regardless of incubation solutions (buffer or MMP‐9). Moreover, the peak shift rates of each cycle were calculated according to Figure 6a (50 ng mL^−1^, top red circles), indicating the average IOP increasing rate was 1.05 ± 0.10 mmHg s^−1^ in the measurements which fitted well with the set rate at 1 mmHg s^−1^ (Figure S17, Supporting Information), suggesting the feasibility of real‐time IOP measurements. The MMP‐9 detection was evaluated by SERS after cycles of IOP monitoring. The reaction time in the simultaneous detections was 1 h for two times as described which was equivalent to the 2 h incubation time used previously. The acquired Raman spectra showed the difference between the sample and negative control (Figure 6c). The ratios of intensities were obtained in the same way that could be used for MMP‐9 concentrations estimation (Figure 6d). As a result, the calculated concentrations derived from the calibration curve based on the ratios of 10 and 50 ng mL^−1^ were estimated as 11 and 48 ng mL^−1^, respectively (blue square and red circle, Figure 6e), suggesting the good accuracy of MMP‐9 detection during the simultaneous IOP monitoring. The independency of MMP‐9 detection was also confirmed by the similar SERS results derived from incubations under two IOP levels (0 and 50 mmHg), where the signal ratios (*I*/*I*_0_) were almost overlapped on the calibration curve (Figure S18, Supporting Information). The results indicated that the SERS measurements were independent without any interference from IOP changes.

The feasibility of dual detections on animals was testified by using portable devices including the reflection and Raman spectrometers (Figure S19a,b, Supporting Information). The IOP monitoring by our smart contact lens showed a similar average IOP as compared to the commercial portable tonometer during 1 h, though the variation was larger (26.7 ± 3.9 and 26.0 ± 1.3 mmHg, respectively, Figure S19c, Supporting Information). The Raman results showed a similar spectrum as obtained under the Raman microscope (bottom black and top blue, Figure S19d, Supporting Information), and a signal drop upon incubating with 50 ng mL^−1^ MMP‐9 (bottom black and red, Figure S19d, Supporting Information). The concentration calculated was around 44 ng mL^−1^. Overall, our smart contact lens had demonstrated the feasible dual‐detection of IOP and MMP‐9 with relatively high accuracy.

## 3 Conclusion

In summary, we have developed a biocompatible, dual‐functional smart contact lens sensor to monitor IOP and detect MMP‐9 in tears, which were the main risk factors related to glaucoma, dry eye, and others. The antiopal structure was applied to continuously monitor the IOP through color changes, without an external power supply, well covering the physiological IOP range. By combining the AuNBs SERS substrate with the structural color contact lens, quantitative analysis of MMP‐9 had been achieved down to 1.29 ng mL^−1^ in tears, enabling the dual detections of IOP and MMP‐9 at a high‐performance level, as compared to other related works for either IOP or MMP‐9 detection (Table S3, Supporting Information). The long‐term direct contact between contact lens and tears also overcame the low tear sampling problem in the case of MMP‐9 detection. Moreover, by using optical methods, the potential damages caused by the electronic components to the eyes could be effectively avoided. The smart contact lenses developed in this work provided a convenient, noninvasive, multifunctional sensing platform for monitoring health and diagnostic biomarkers in human tears, and paved a way for integrating more functions into contact lenses as wearable health‐monitoring devices in the future.

## 4 Experimental Section

### Materials and Characterization

2‐Hydroxyethyl methacrylate (HEMA, 99%), ethylene glycol dimethacrylate (EGDMA, 98%), 2‐hydroxy‐2‐methyl‐1‐phenyl‐1‐propanone (HMPP, 97%) were all purchased from Shanghai McLean Biochemical Technology Co., Ltd. The monodisperse PS microspheres with a diameter of 500 nm were purchased from Shanghai So‐Fe Biomedicine Co., Ltd. Monodispersed SiO_2_ microspheres with different diameters (180, 240, 300 nm) were purchased from Nanjing Nanorainbow Biotechnology Co., Ltd. Tris‐HCl (1×), PBS (1×, pH 7.4), Recombinant human MMP‐9, and cell culture products were all purchased from Sigma‐Aldrich. HF (AR, 40%) was purchased from Aladdin Chemical Company Co., Ltd. The p‐aminophenyl mercuric acetate was provided by Genmed Scientifics Inc. The Tamra‐modified peptide (Tamra‐Lys‐Pro‐Leu‐Gly‐Leu‐Dap(Dnp)‐Ala‐Arg‐Cys‐NH_2_, purity > 98%, MW = 1521.0) and the fluorescent peptide (MOCAc‐Lys‐Pro‐Leu‐Gly‐Leu‐Dap(Dnp)‐Ala‐Arg‐NH_2_, purity > 98%, MW = 1221.6) were both purchased from Shanghai Apeptide Co., Ltd. 293T cells were purchased from Procell Life Science & Technology Co., Ltd. CCK‐8 was obtained from Dojindo Molecular Technologies, Inc. Deionized water was purified by Milli‐Q Ultrapure Water System (Millipore).

The polymerization process of the monomer solution was done by a UV LED curing system (UVATA Technology Co., Ltd.) for 10 min. The morphology and microstructure of contact lenses embedded with photonic crystal templates before and after etching of the templates were characterized using field emission scanning electron microscopy (SU8010, HITACHI), and the structure of AuNBs was also characterized. The reflectance spectra of the structural color were measured by a fiber‐optic spectrometer (Idea Optics). The water loss (*W*_L_) of the contact lenses was obtained by measuring the wet weight (*W*_t_) and dry weight (*W*_D_) of the contact lenses and was calculated by *W*_C_ = *W*_D_ / *W*_t_ × 100%. The SERS spectrum was obtained by confocal Raman microscope (InVia, Renishaw) with an excitation light wavelength of 632.8 nm (He‐Ne laser, 17 mW). The fluorescence intensity of fluorescent peptides was tested using a full‐wavelength microplate reader with excitation/emission wavelengths at 322/381 nm (Thermo Scientific Varioskan LUX). The 293T cells were cultured in Dulbecco's modified Eagle's medium (DMEM, Sigma‐Aldrich) supplemented with 10% fetal bovine serum and 1% penicillin‐streptomycin (100 U mL^−1^). The cytotoxicity of the contact lens sensor was detected by quantifying the cell proliferation activity of cells cultured for 3 consecutive days using the CCK‐8. The slit lamp microscope (SLM‐7E, Chongqing Kanghua Ruiming, China) was employed for imaging animal eyes.

### Preparation of Structural Color Contact Lenses

To prepare structural color contact lenses, it was first necessary to self‐assemble photonic crystals in the bottom mold of the contact lens mold. The suspensions containing monodisperse SiO_2_ particles with different average sizes (180, 240, 300 nm) were dropped into each mold and then dried in an oven at 80 °C. Due to the coffee ring effect, the ringlike photonic crystal template can be formed in the mold. The SiO_2_ nanoparticles deposited in the center of the mold were carefully removed with 2% HF solution. After forming photonic crystal templates with different colors, precursor solutions were added containing HEMA (1 mL) as a monomer, EGDMA (15 µL) as a crosslinker and HMPP (9.5 µL) as a photoinitiator into each mold and left under vacuum for 10 min to infiltrated into the voids of the photonic crystal templates. Then the top mold was pressed into the bottom mold to form the shape of the lens, and the precursor solution was photopolymerized for 10 min with a UV curing lamp. The polymerized lens was carefully removed from the mold and then immersed into the 4% HF solution to remove the SiO_2_ template. Finally, the lens was repeatedly washed at least 5 times with deionized water and saline to remove the residual HF, and finally, the structural color contact lens with an antiopal structure was obtained.

### Ex Vivo Test for the Contact Lens Sensor Using a Porcine Eye

The enucleated porcine eyes were used for ex vivo experiments because they were similar in size to human eyes. A syringe pump and a pressure sensor were connected on both sides of the porcine eyeball to inject constant pressure into the eyeball and to confirm the internal pressure. The pressure variation was controlled in the range of 0–50 mmHg, and the rate of pressure change was controlled at 1 mmHg s^−1^. A cover was put over the porcine eye with water drops around it to maintain the humidity (46%, 20 °C) in order to avoid the interference of the IOP monitoring caused by the water loss. In addition, a fiber optic spectrometer was used to monitor the structural color changes in real‐time.

### Preparation of 2D‐PS Opal Template

First, the PS nanoparticles were dispersed in a mixture of water and ethanol at a volume ratio of 1:1. The Si wafer was cut into pieces with a size of 2 × 2 cm^2^. Then, the substrates were cleaned with acetone, ethanol, and deionized water successively for 30 min. Finally, using the piranha solution to remove residual impurities on Si wafers. To make their surface hydrophilic, they were treated with O_2_ plasma cleaner for 5 min. 100 µL of deionized water was taken onto the treated Si wafer, and after the water surface was stabilized, PS suspension was slowly added dropwise until the water surface was covered by a PS film. At this point, the deionized water under the PS film was carefully absorbed with a paper towel. And when the water was thoroughly dried, a monolayer of 2D‐PS opal was formed on the Si wafer.

### Preparation of AuNBs SERS Substrate

The Au‐coated opal template was obtained by depositing a 150 nm Au layer on the PS opal template using a high vacuum electron beam evaporation coater (TEMD600). Subsequently, the Au‐coated opal template was transferred from the Si wafer using water‐soluble tape. At this time, the Au layer was adhered to the water‐soluble tape, while the PS layer was exposed. Then, 100 µL of the PS‐toluene solution was spin‐coated on the above tape and dried to form a film. The water‐soluble tape was then transferred to a petri dish filled with water and waited for the water‐soluble tape to dissolve. Once the tape was dissolved, the SiO_2_/Si wafers could be used to pick up the PS‐Au template floating on the water surface. Next, the SiO_2_/Si wafer was placed in chloroform overnight to obtain the AuNBs structure.

### Preparation of “SERS‐Structural Color” Contact Lens

First, the SiO_2_/Si wafer with AuNBs substrate was placed at 4% HF. After 5 min, the AuNBs substrate was carefully transferred to a 6 cm petri dish filled with water before leaving the wafer completely. Then, the AuNBs substrate was able to float completely to the water surface. Subsequently, the substrate was picked up by the outer surface of the structural color contact lens, where the position of the AuNBs substrate on the contact lens could be controlled by ourselves. After waiting for the water to dry on the contact lens surface, the lenses were repeatedly washed at least 5 times with deionized water and saline solution to remove residual HF. Finally, the lenses were kept in saline solution for later use.

### Preparation of Peptide‐Modified AuNBs SERS Substrate

20 µL (10 × 10^−6^ m) Tamra‐pep solution was dropped onto the AuNBs substrate on a dry contact lens and stored at room temperature for 30 min to modify the substrate. Subsequently, the substrate surface was rinsed extensively with deionized water 3 times to remove free peptides.

### SERS Detection of MMP‐9 Enzyme

The Tamra‐pep modified AuNBs substrate was incubated with 100 µL different concentrations of activated MMP‐9 at 37 °C, 2 h. Subsequently, the surface of the substrate was rinsed with deionized water to wash away the residual MMP‐9 and the cleaved peptide fragments. SERS spectra and SERS imaging pictures were obtained by the confocal Raman microscope with an excitation light wavelength of 632.8 nm (with power of 0.836 mW, the integration time *t* = 3 s) and a 50× long objective lens. For the detections in tears, the collected tear samples were centrifuged at 10000 r for 5 min and 50 µL of supernatant was mixed 1:1 with Tris‐HCl buffer. The obtained mixture was placed in a refrigerator at 4 °C until further usage.

### Specificity Test for MMP‐9

The peptide‐modified AuNBs substrates were incubated with 100 µL different enzymes (1 µg mL^−1^ MMP‐9, 1 µg mL^−1^ trypsin, and 1 µg mL^−1^ lysozyme) at 37 °C, 2 h, respectively. Subsequently, their Raman spectra were obtained by the confocal Raman microscope and their Raman intensities were compared.

### Fluorescence Detection of MMP‐9 Enzyme

50 µL (10 × 10^−6^ m) fluorescent peptide was mixed with 50 µL MMP‐9 solution with different concentrations, and the fluorescence intensity was recorded by the microplate reader, and the kinetics curves of fluorescence were obtained. The total time was 30 min, and the values were read every 3 s.

### Collection of Human Tears

A 10 µL glass capillary tube was used to collect the tears. The capillary tube was placed in the lateral tear meniscus and avoided contact with the conjunctiva of the eye as much as possible. When the capillary was filled to about 1/4 of its full length, the tears could be transferred to an Eppendorf centrifuge tube. The collection process was repeated until sufficient tears had been collected. All volunteers were gave written and informed consent before participation in the experiment.

### Cell Viability Assay

Cells were cultured in a humidified incubator at 37 °C with 5% CO_2_. Before further incubation, structural color contact lenses containing AuNBs were immersed in 75% alcohol for 2 h in conjunction with UV irradiation to kill bacteria and viruses. Subsequently, contact lenses were rinsed 3 times with PBS and immersed in DMEM overnight. Cells were inoculated on the surface of sterilized contact lenses and in blank wells of 96‐well cell culture plates (control) and incubated for 1–3 days. The number of living cells in the experimental and control groups was quantified by the CCK‐8 after removal of the contact lenses, in which the O.D. values at a wavelength of 450 nm were tested using a microplate reader. In this case, the magnitude of absorbance was related to the number of living cells.

### In Vivo Biocompatibility Test

All in vivo studies were conducted according to the guidelines for care and use of laboratory animals and approved by the ethics review committee of the Institute of Animal Care and Use Committee of Wenzhou Institute, University of Chinese Academy of Sciences (WIUCAS21101839). As for in vivo biocompatibility testing of smart contact lenses, slit lamp examinations were performed for rabbit eyes. First, smart contact lenses and commercial contact lenses (Clariti 1 day, from CooperVision, Inc.) were worn on the rabbit eyes for 8, 16, and 24 h, respectively. Subsequently, removing the worn contact lenses, and 3 µL of 1% sodium fluorescein in saline solution were applied to the lower conjunctival sac to evaluate the ocular surface with a slit lamp (cobalt‐blue filter, magnification 10×).

### In Vivo IOP and SERS Measurements

To prove the concept of in vivo measurements, the portable fiber‐optic spectrometer (Idea Optics) and a portable fiber‐based Raman spectrometer (QEpro, Ocean Optics, laser wavelength of 785 nm) were simultaneously applied on the top of the eye of a living rabbit wearing a contact lens for the measurement of IOP and MMP‐9 changes (Figure S19a,b, Supporting Information). As for comparison, a commercial tonometer (TONO‐PEN AVIA, Reichert) was applied for the monitoring of the rabbit IOP every 5 min. For the measurement of MMP‐9, the Raman spectra were taken before and after applying of 50 ng mL^−1^ MMP‐9 on the contact lens and incubated for 2 h.

### Statistical Analysis

The IOP data were preprocessed accordingly by normalization. All data were expressed as the mean ± s.d. All quantitative experiments were repeated at least three times. Student's *t*‐test was utilized for single comparisons. All statistical analyses were performed using GraphPad Prism 8 software. The statistical significance was set at *p* = 0.05 (*p < 0.05, **p < 0.01, ***p < 0.001).

## Conflict of Interest

The authors declare no conflict of interest.

## Author Contributions

Y.Y. and C.G. contributed equally to this work. Y.W., X.L., and J.Q. conceived the idea and designed the experiments. Y.Y. and Y.G. performed most of the experiments. Y.Y. and X.L. did the data analysis. X.L. and Y.Y. wrote the manuscript with the support of Y.W. and J.Q. All authors discussed the results and commented on the manuscript.

## Supporting information

Supporting Information

Supplemental Video 1

## Data Availability

The data that support the findings of this study are available from the corresponding author upon reasonable request.
