# Supplementary material for: Smart Contact Lens with Dual‐Sensing Platform for Monitoring Intraocular Pressure and Matrix Metalloproteinase‐9
Source: Adv Sci (Weinh). 2022 Feb 23;9(12):2104738. doi: 10.1002/advs.202104738 (PMC9036001; doi:10.1002/advs.202104738)
Supplement: Supplementary file 1 — Supporting Information [file ADVS-9-2104738-s002.pdf]

ADVANCED SCIENCE
Open Access

Supporting Information

for *Adv. Sci.*, DOI 10.1002/advs.202104738

Smart Contact Lens with Dual-Sensing Platform for Monitoring Intraocular Pressure and Matrix Metalloproteinase-9

Ying Ye, Yuancai Ge, Qingwen Zhang, Meiling Yuan, Yu Cai, Kang Li, Yang Li, Ruifeng Xie, Changshun Xu, Danfeng Jiang, Jia Qu, Xiaohu Liu* and Yi Wang**

Supporting Information

Smart Contact Lens with Dual-sensing Platform for Monitoring Intraocular Pressure and Matrix Metalloproteinase-9

Ying Ye, Yuancai Ge, Qingwen Zhang, Meiling Yuan, Yu Cai, Kang Li, Yang Li, Ruifeng Xie, Changshun Xu, Danfeng Jiang, Jia Qu *, *Xiaohu Liu* *, *Yi Wang* *

Y. Ye, Dr. Y. Ge, Dr. Q. Zhang, M. Yuan, Y. Cai, K. Li, Y. Li, R. Xie, C. Xu, Dr. D. Jiang, Pro. J. Qu, Dr. X. Liu, Pro. Y. Wang

School of Ophthalmology and Optometry, Eye Hospital, School of Biomedical Engineering Wenzhou Medical University

Wenzhou 325027, P. R. China

E-mail: wangyi@wiucas.ac.cn; liuxiaohu@wmu.edu.cn; qujia@eye.ac.cn

Y. Ye, Dr. Y. Ge, Dr. Q. Zhang, M. Yuan, Y. Cai, K. Li, Y. Li, R. Xie, C. Xu, Dr. D. Jiang, Dr. X. Liu, Pro. Y. Wang

Wenzhou Institute, University of Chinese Academy of Sciences

Wenzhou 325001, P. R. China

R. Xie, Pro. Y. Wang

School of Opto-Electronic Engineering

Changchun University of Science and Technology

Changchun 130022, P.R. China.

Keywords: smart contact lens, dual-sensing platform, intraocular pressure, matrix metalloproteinase-9, SERS

Table of Content

Comparison of the contact lenses (Table S1).

Preparation and characterization of structure-color contact lenses related to IOP monitoring (Figure S1-S5).

Preparation and characterization of SERS substrates (Figure S6-S9).

Quantitative analysis of MMP-9 with peptide substrates (Figure S10-S15 and Table S2).

Biocompatibility and dual detections of IOP and MMP-9 (Figure S16-S19).

Comparison of the performances in literature (Table S3).

Table S1. Comparison of our dual-functional smart contact lens with other commercial contact lenses.

Products	Moisture content	Oxygen transmission (Dk) (cm^2/s) ($mLO_2/(mL \times mmHg)$)	Transmissivity
**This work**	38%	8.4×10^{-11}	≥ 85%
**Biomedics 38**	38%	8.4×10^{-11}	≥ 90%
**SofLens 38**	38.6 ± 2%	8.4×10^{-11}	≥ 92%

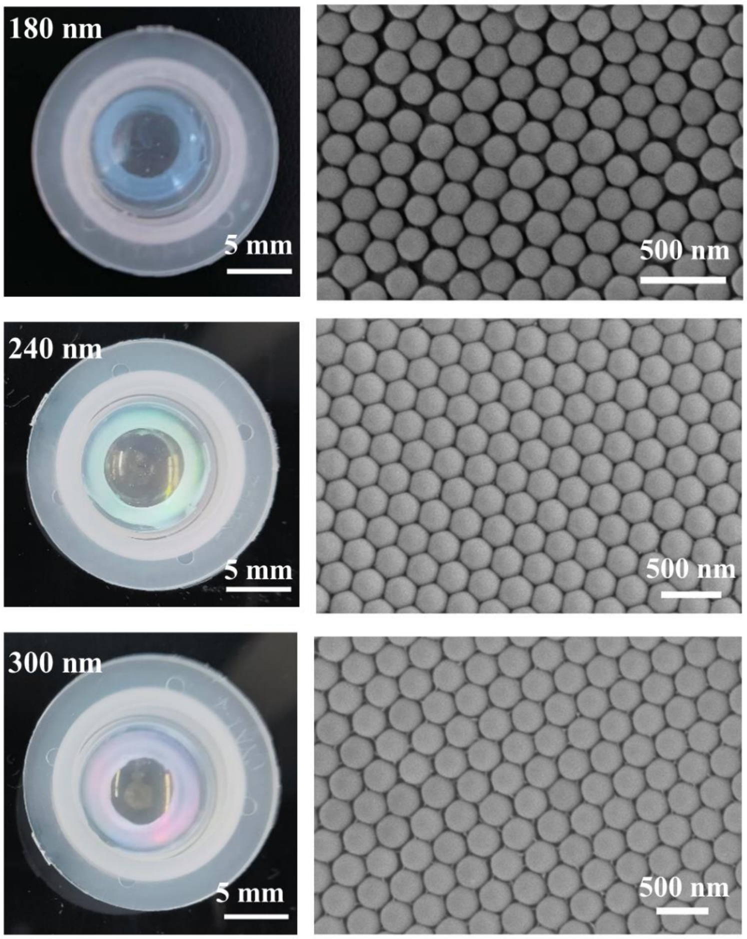

Figure S1. Photographs of photonic crystals formed by SiO_2 nanoparticles with different particle sizes (180 nm, 240 nm, 300 nm) in contact lens molds (left) and their corresponding SEM images (right). Scale bars: 5 mm (left) and 500 nm (right).

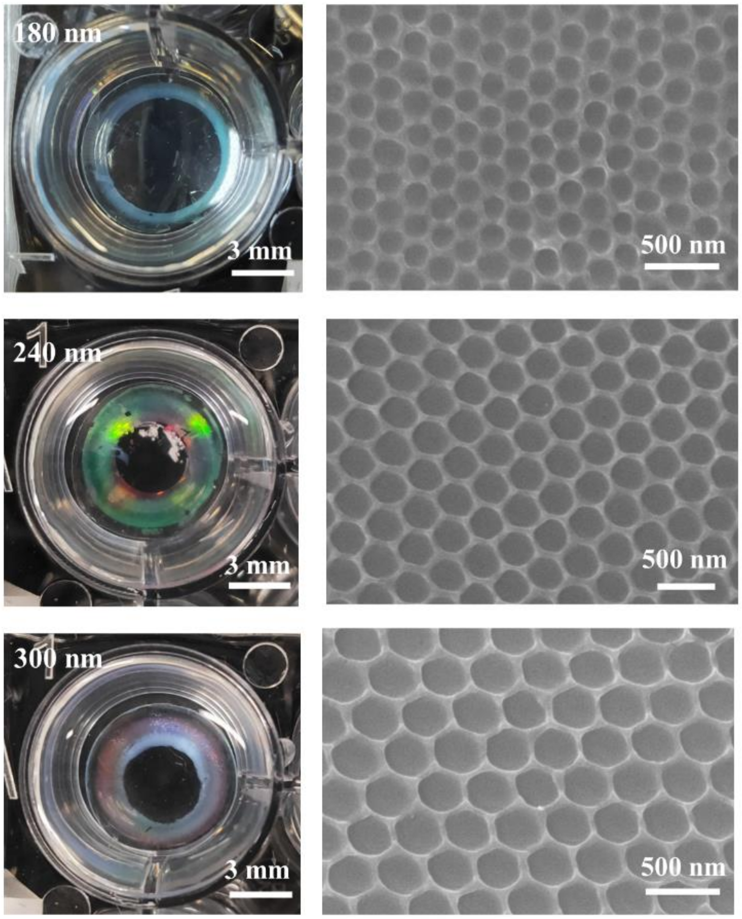

Figure S2. Photographs of structural color contact lenses (in water) after removing the photonic crystal templates in Figure S1 by HF (left) and their corresponding SEM images (right). Scale bars: 3 mm (left) and 500 nm (right).

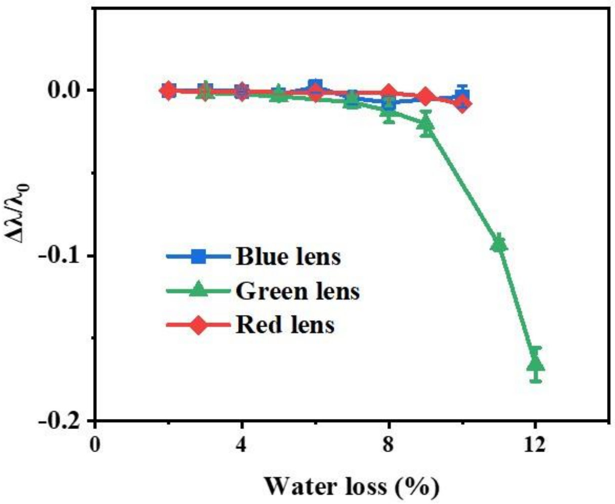

Figure S3. The relationship between the shift ratio of reflection spectra and the water loss with three different structural color contact lenses (in the air) prepared in Figure S2. All data were presented as mean ± s.d. (n > 3).

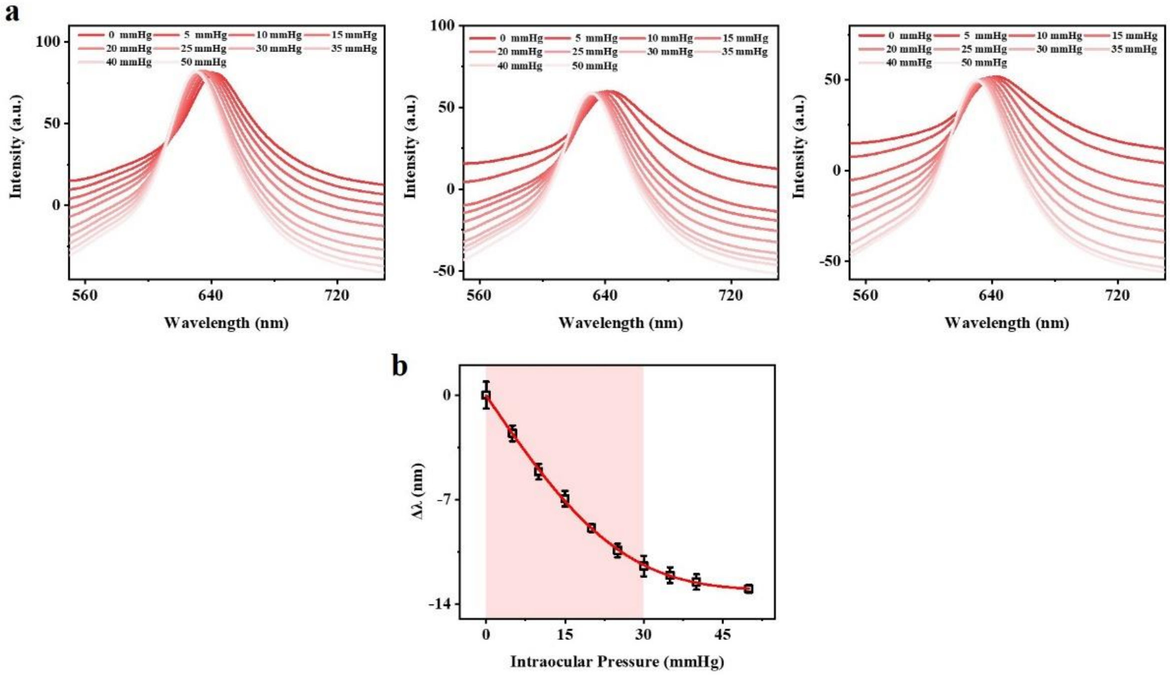

Figure S4. The representative spectra (a) and derived spectral peak shifts (b) of green structural color when IOP changes within the range of 0 - 50 mmHg. The red area in (b) referred to IOP in the physiological range of 0 - 30 mmHg as shown in Figure 3e. All data were presented as mean ± s.d. (n > 3).

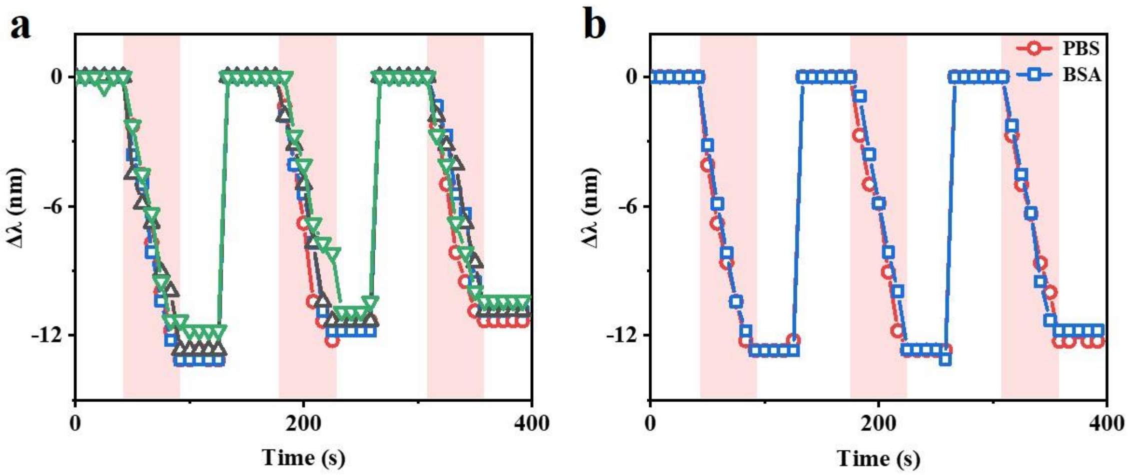

Figure S5. a) The cyclic real-time tests on 4 different smart contact lenses for the IOP monitoring. b) The cyclic real-time tests on smart contact lenses for IOP monitoring in PBS (red) and in PBS with 10 mg/mL BSA (blue). The red shaded region referred to IOP increment by injection of fluids.

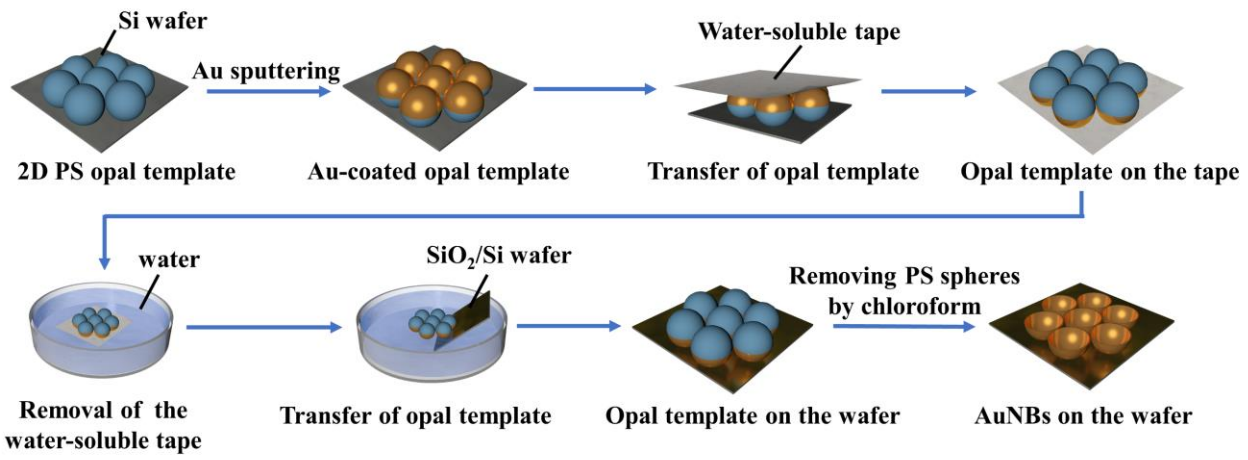

Figure S6. Schematic diagram of the preparation process of AuNBs used in the smart contact lens.

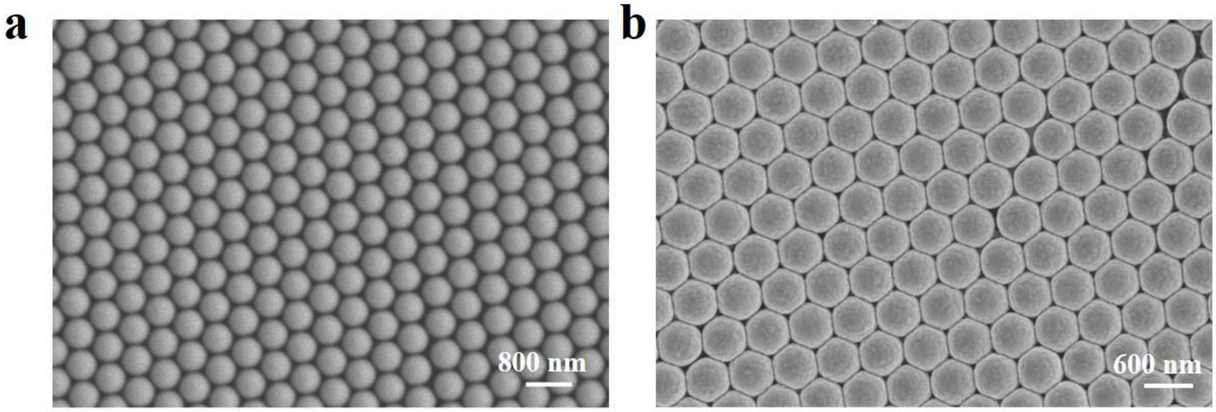

Figure S7. a) SEM image of periodically ordered, dense PS monolayer on Si substrate. Scale bar: 800 nm. b) SEM image of the 150 nm-thick Au/PS monolayer on the Si substrate. Scale bar: 600 nm.

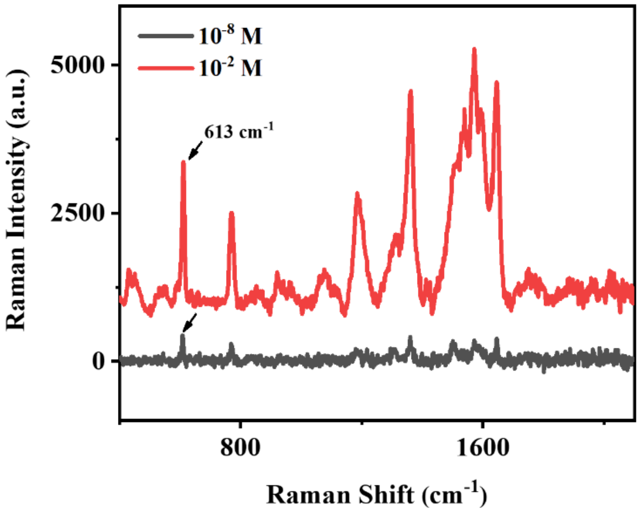

Figure S8. The Raman intensities at 613 cm^{-1} of 10^{-2} M R6G on glass substrate (2026.7 a.u., red line) and 10^{-8} M R6G on AuNBs SERS substrate (627.1 a.u., black line) for the calculation of enhancement factor according to the following equation:

$$EF = \frac{I_{SERS} \times C_{bare}}{I_{bare} \times C_{SERS}}$$

The C_{bare} and I_{bare} were the concentration of R6G and the Raman intensity acquired on the glass substrate, while the C_{SERS} and I_{SERS} were the concentration of R6G and the Raman intensity acquired on AuNBs substrate, respectively.

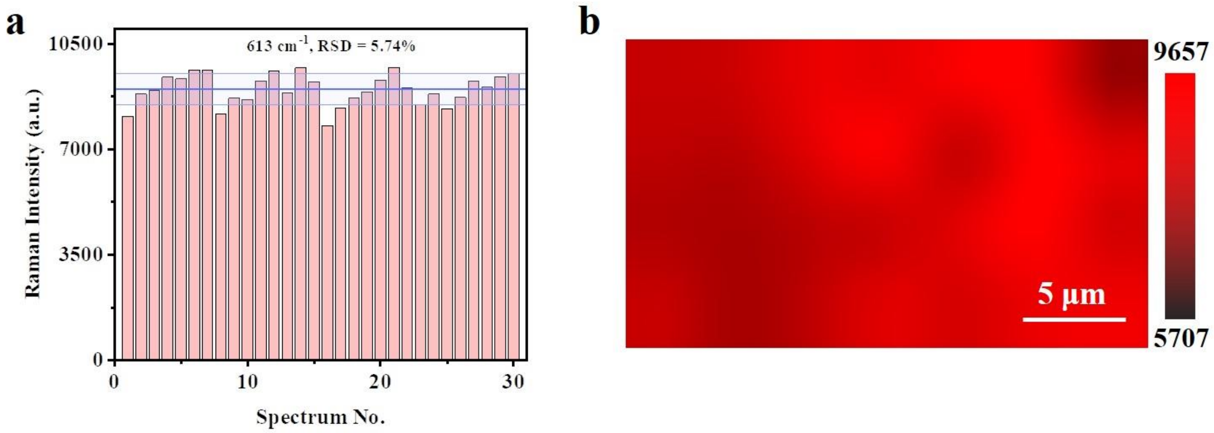

Figure S9. a) The Raman intensities of 30 spectra at 613 cm^{-1} (the average value was plotted with the violet line, and the lilac region indicated the variation of ± 5.74%). b) The Raman mapping of 10^{-7} M R6G with a rectangle of 19 × 11 μm^2 on the Au/PS monolayer substrate. Scale bar: 5 μm.

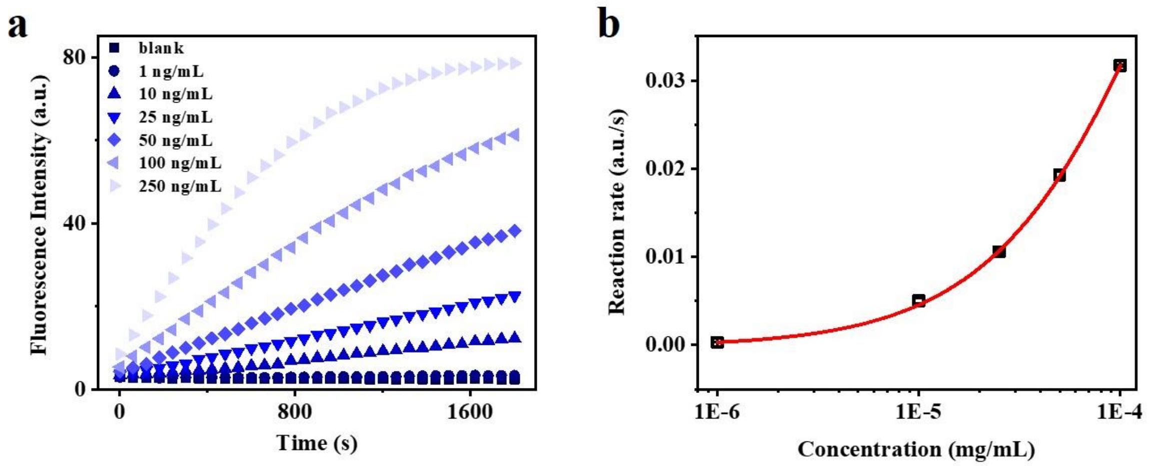

Figure S10. a) The fluorescence kinetics curves of the fluorescent peptides cleaved by MMP-9 at different concentrations (1 ng/mL, 10 ng/mL, 25 ng/mL, 50 ng/mL, 100 ng/mL, and 250 ng/mL). b) The fitting curve plotted according to the fluorescence kinetics in a). All data were presented as mean ± s.d. (n > 3).

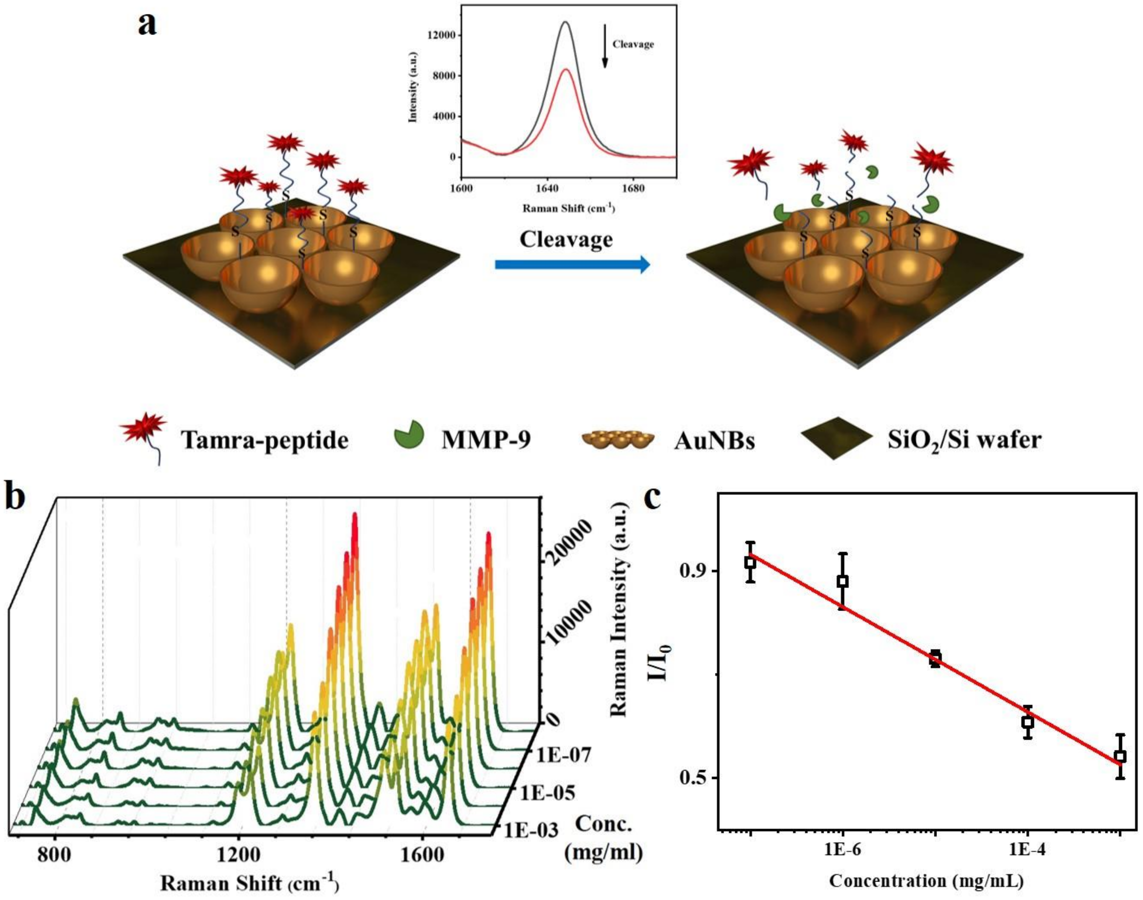

Figure S11. a) The schematic diagram of the Tamra-pep cleavage by MMP-9 at the surface of AuNBs on the SiO_2/Si wafer. b) Raman spectra of Tamra-pep modified at the surface of AuNBs substrate on the SiO_2/Si wafer after different concentrations of MMP-9 treatment (0, 0.1 ng/mL, 1 ng/mL, 10 ng/mL, 100 ng/mL, 1 μg/mL). c) The corresponding linear calibration curve (R^2 = 0.969) was plotted according to the Raman spectral responses in b). All data were presented as mean ± s.d. (n > 3). The LOD of MMP-9 was estimated to be 0.29 ng/mL according to the fitted line with 3 times of standard deviation (σ_{wafer} = 0.885).

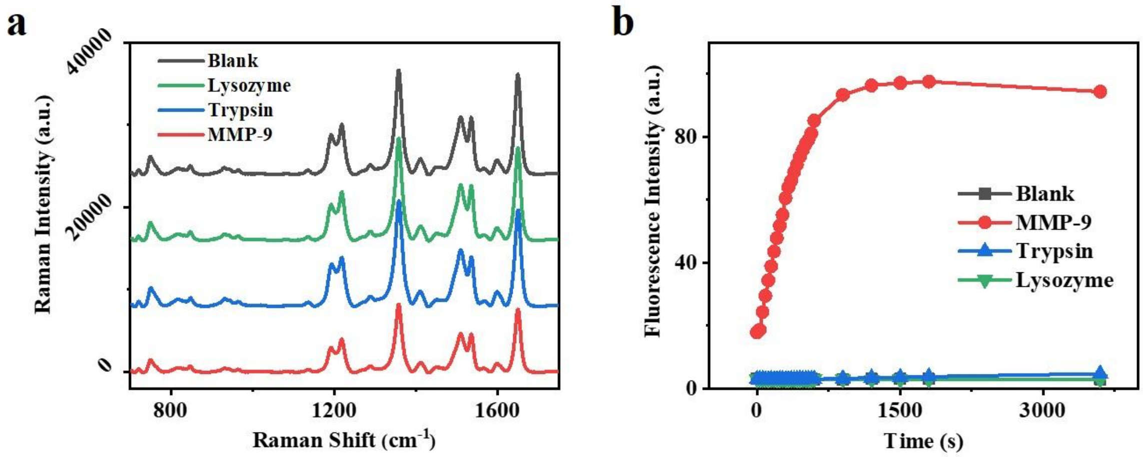

Figure S12. a) Raman spectra of Tamra-pep modified at the surface of AuNBs substrate after treatment of MMP-9 (1 μg/mL, red), trypsin (1 μg/mL, green), lysozyme (1 μg/mL, blue) and Tris-HCl solution (black). b) Fluorescence kinetic curves of fluorescent peptides cleaved by MMP-9 (1 μg/mL, red), trypsin (1 μg/mL, green), lysozyme (1 μg/mL, blue) and Tris-HCl solution (black) (n > 3).

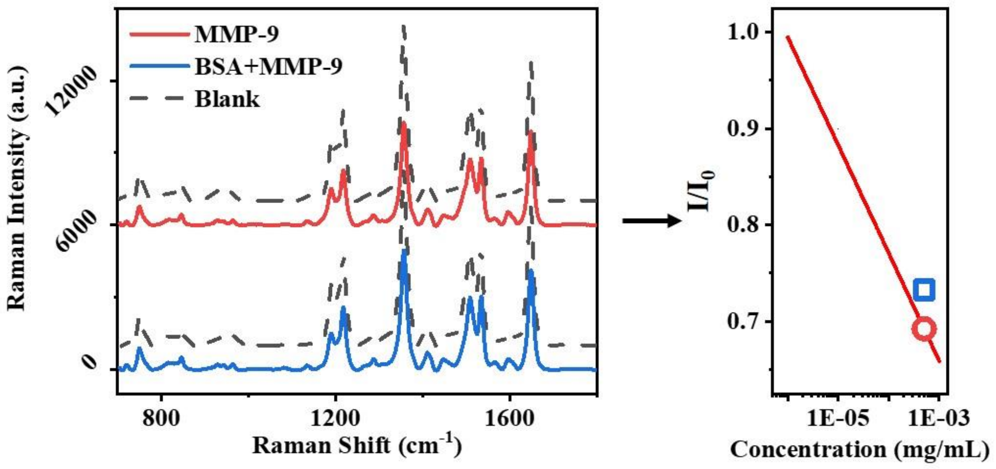

Figure S13. The Raman spectra after 2 hours incubations with 500 ng/mL MMP-9 only (red, left), and MMP-9 mixed with 9 mg/mL BSA (blue, left). Right: The ratios of Raman intensities of MMP-9 samples to that of blank samples at 1648 cm^{-1} were added on the calibration curve in Figure 5c.

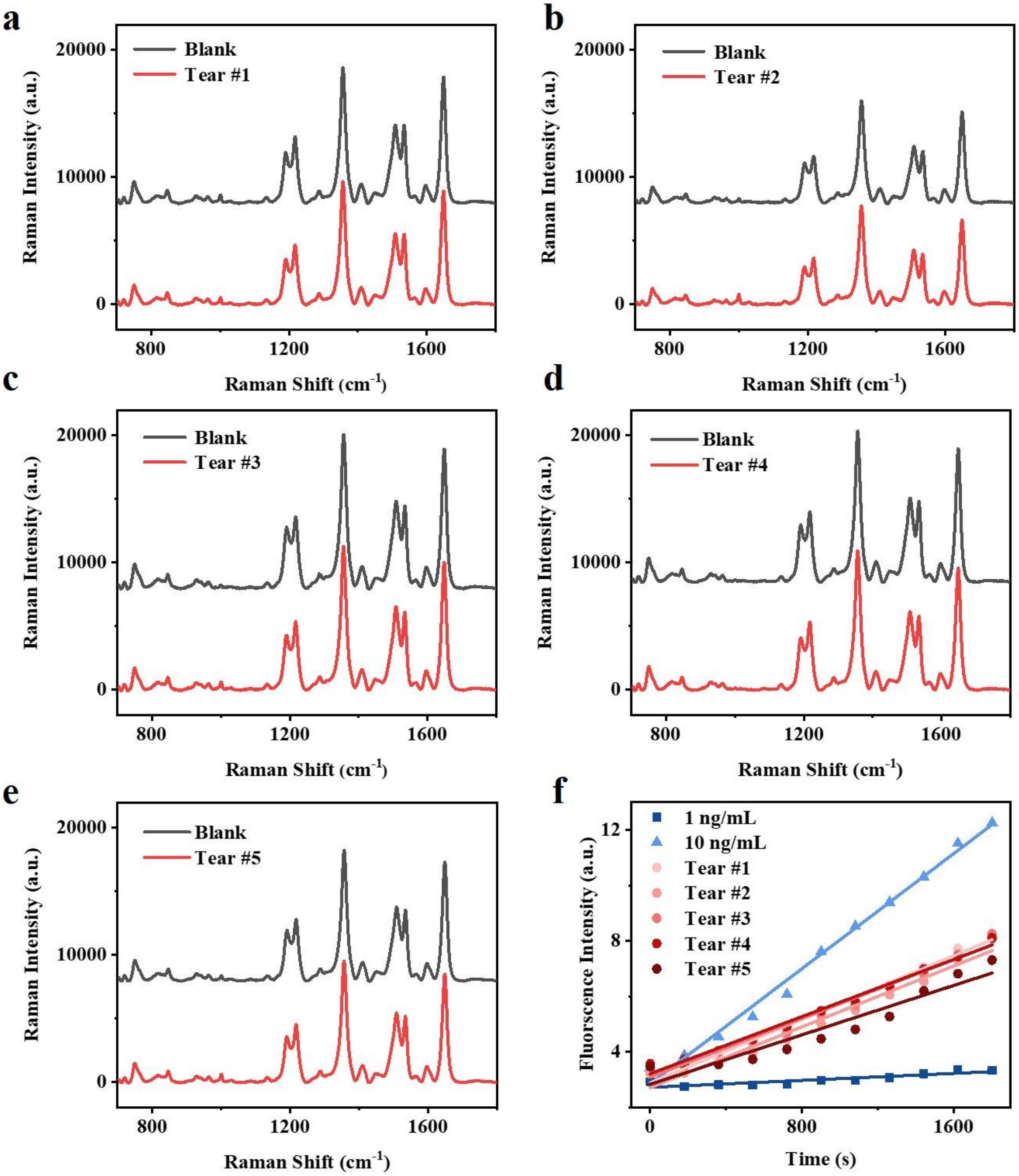

Figure S14. a-e) Raman spectra of Tamra-pep before and after cleavage by the intrinsic MMP-9 in five samples of tears, respectively (red line: tear samples; black line: negative controls). f) The fluorescence kinetics curves of the fluorescent peptides cleaved by the intrinsic MMP-9 in five samples of tears (red lines), compared to the 1 ng/mL and 10 ng/mL MMP-9 (blue lines).

Table S2. Comparison of the intrinsic MMP-9 levels in five different tear samples measured by SERS contact lens and the fluorescent assay.

No.	Conc. (ng/mL) by SERS	Conc.(ng/mL) by Fluo.	Ratios of Diff.
**1**	6.33	5.63	0.12
**2**	4.66	5.05	-0.08
**3**	4.83	5.67	-0.15
**4**	5.09	5.90	-0.14
**5**	5.00	5.43	-0.08

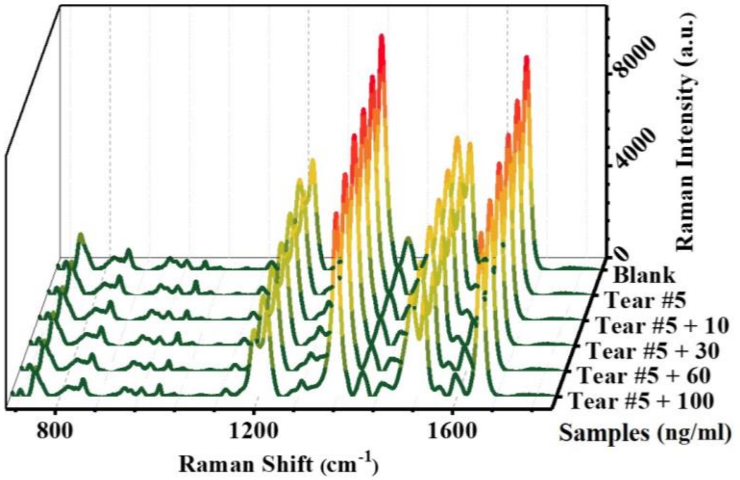

Figure S15. The Raman spectra of Tear #5 samples by spiking different concentrations of MMP-9 (0, 10 ng/mL, 30 ng/mL, 60 ng/mL, and 100 ng/mL).

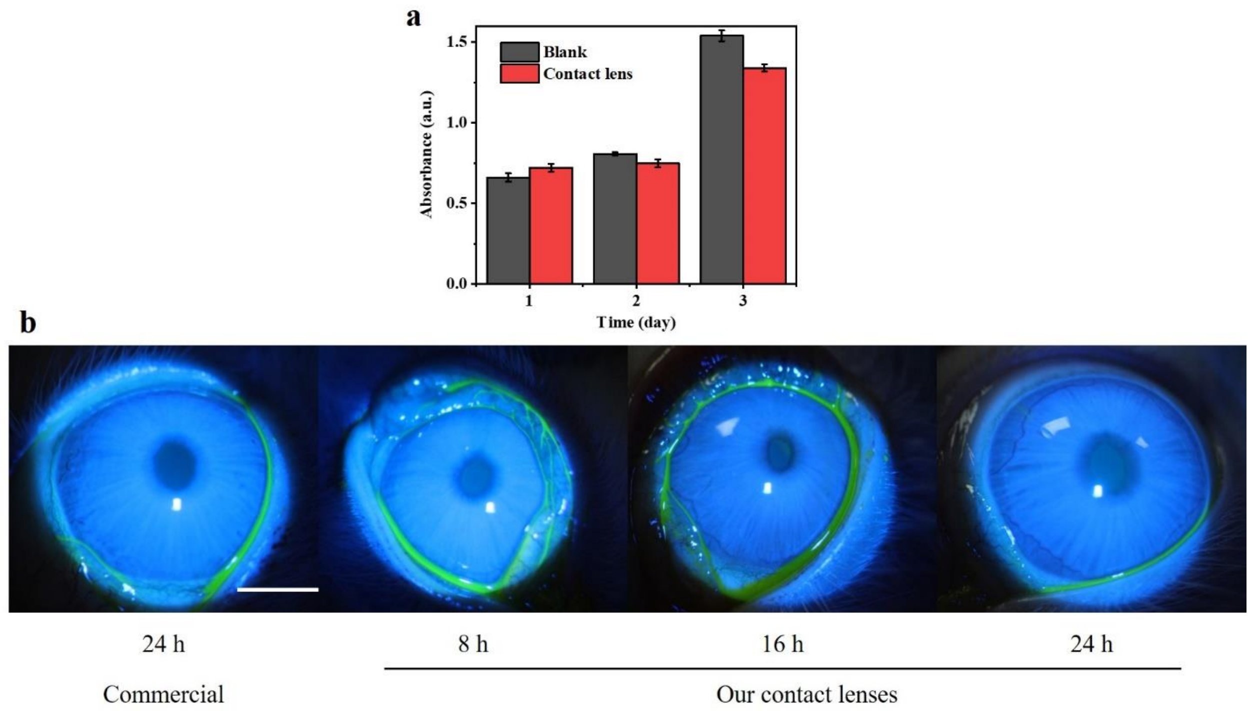

Figure S16. a) The O.D. values of the CCK-8 tests for 293T cells cultured on the structural color contact lenses (red) and controls (black) with different culturing times. All data were presented as mean ± s.d. (n > 3). b) Fluorescent images of the in vivo biocompatibility test on rabbits, wearing the commercial contact lens for 24 h, our contact lenses for 8 h, 16 h, and 24 h. Scale bar: 5 mm. The images of the commercial one and our contact lens for 24 h are presented in Figure 6a as well.

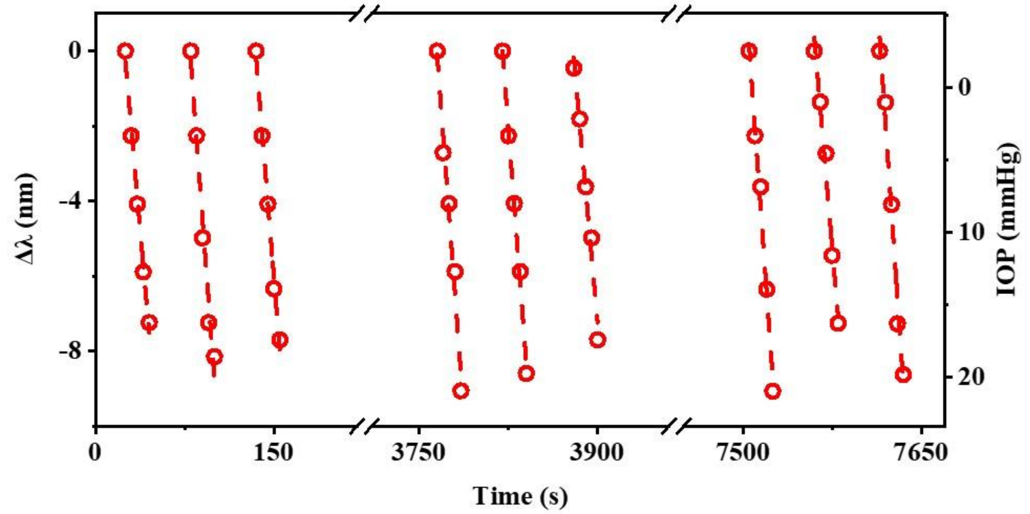

Figure S17. The wavelength shifts coordinated with IOP measured in the dual detections by using smart contact lenses (in 50 ng/mL MMP-9 solution). The slopes of fitted broken lines indicated the changing rates for each cycle.

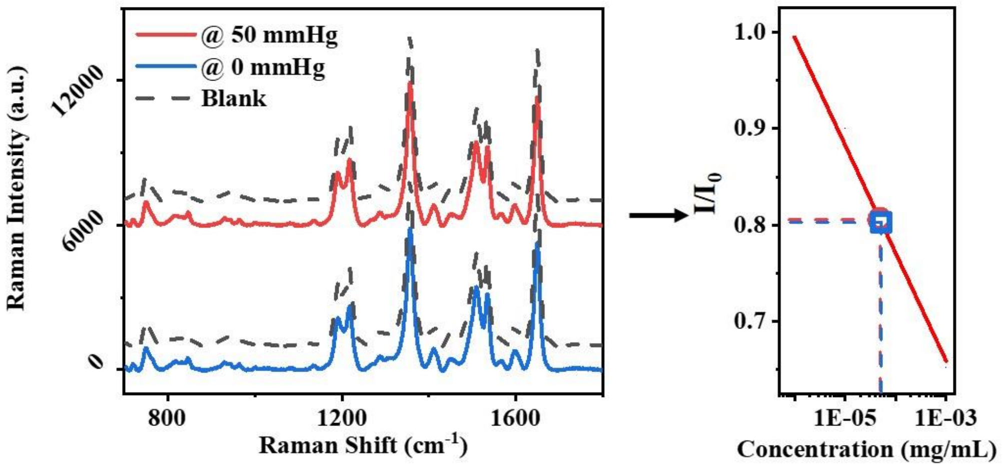

Figure S18. The Raman spectra after 2-hours incubations with 50 ng/mL MMP-9 under 50 mmHg (red, left) and 0 mmHg (blue, left) IOPs of porcine eyes. Right: The ratios of Raman intensities of MMP-9 samples to that of blank samples at 1648 cm^{-1} were added on the calibration curve in Figure 5c.

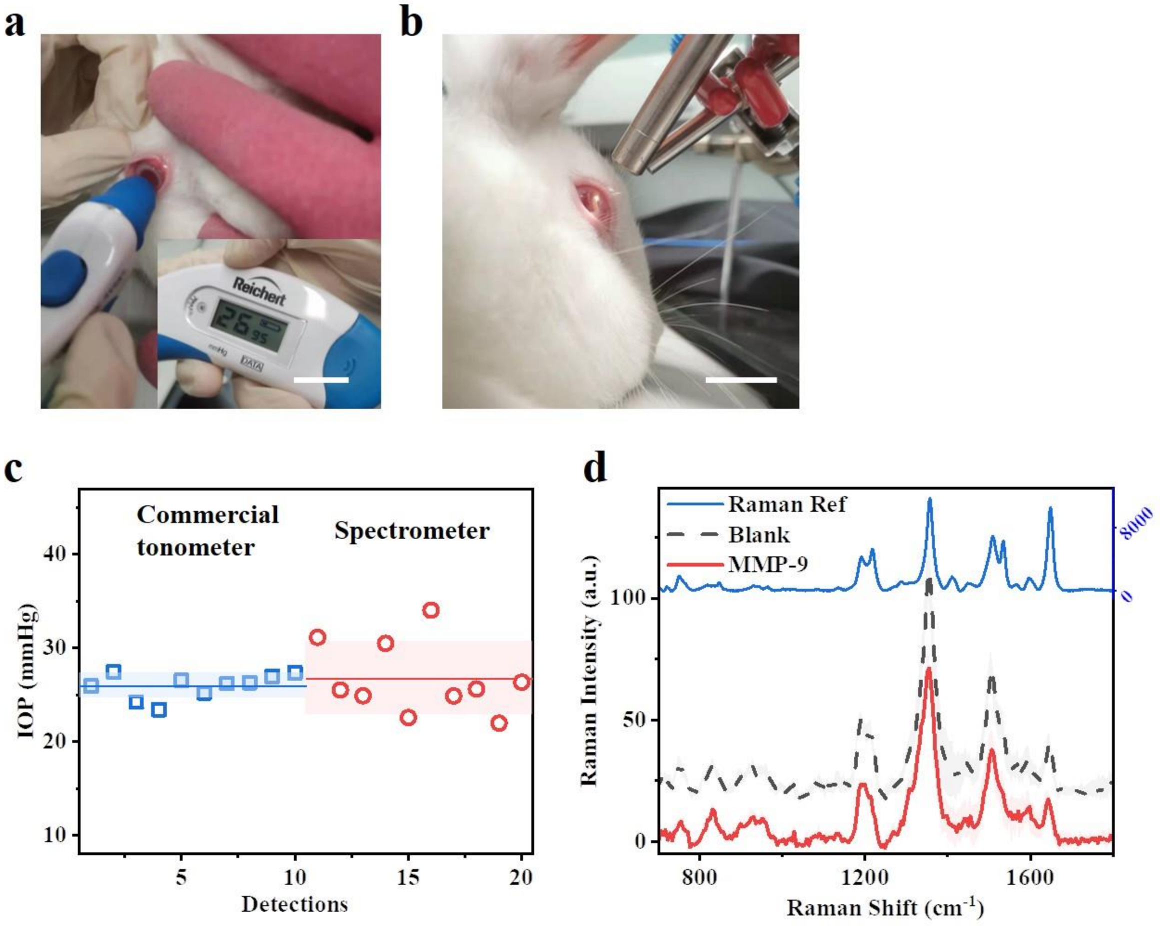

Figure S19. a) Photograph of a rabbit for the measurement of IOP with a commercial tonometer (inset: TONO-PEN AVIA®, Reichert). Inset scale bar: 20 mm. b) Photograph of the dual tests of IOP (right probe of spectrometer) and MMP-9 on a rabbit (left probe of Raman device). Scale bar: 20 mm. c) The IOPs monitoring obtained from a commercial tonometer (blue on the left) and our setup (red on the right). d) The MMP-9 SERS detection by a portable Raman device before (bottom black) and after (bottom red) 50 ng/mL MMP-9 cleavage, with the reference Raman spectrum (top blue) obtained by confocal Raman microscope ($n > 3$).

Table S3. Comparison of performances in related works.

Reference	Biomarker	Working Principle	Sensitivity/LOD	Detection Range
[1]	IOP	Capacitive	2.64 MHz mm Hg^{-1}	0 - 50 mmHg
[2]	IOP	Bragg Diffraction	0.23 nm $mmHg^{-1}$	10 - 60 mmHg
[3]	IOP	Piezo-resistive	3.17 mV $mmHg^{-1}$	16 - 32 mmHg
[4]	IOP	Piezo-resistive	0.05% $mmHg^{-1}$	0 - 30 mmHg
**This work**	IOP	Bragg Diffraction	0.38 nm $mmHg^{-1}$	0 - 30 mmHg
	MMP-9	SERS	0.90 ng ml^{-1}	1 - 1000 ng ml^{-1}
[5]	MMP-9	Field Effect Transistor	0.74 ng ml^{-1}	1 - 500 ng ml^{-1}
[6]	MMP-9	Fluorogenic	369.84 ng ml^{-1}	0 - 1840 ng ml^{-1}

[1] J. Kim, M. Kim, M. S. Lee, K. Kim, S. Ji, Y. T. Kim, J. Park, K. Na, K. H. Bae, H. Kyun Kim, F. Bien, C. Young Lee, J. U. Park, Nat Commun 2017, 8, 14997, https://doi.org/10.1038/ncomms14997.

[2] B. Maeng, H.-k. Chang, J. Park, Lab Chip 2020, https://doi.org/10.1039/c9lc01268k.

[3] Z. Liu, G. Wang, C. Ye, H. Sun, W. Pei, C. Wei, W. Dai, Z. Dou, Q. Sun, C. T. Lin, Y. Wang, H. Chen, G. Shen, Advanced Functional Materials 2021, https://doi.org/10.1002/adfm.202010991.

[4] J. Kim, J. Park, Y.-G. Park, E. Cha, M. Ku, H. S. An, K.-P. Lee, M.-I. Huh, J. Kim, T.-S. Kim, D. W. Kim, H. K. Kim, J.-U. Park, Nature Biomedical Engineering 2021, https://doi.org/10.1038/s41551-021-00719-8.

[5] J. Jang, J. Kim, H. Shin, Y.-G. Park, B. J. Joo, H. Seo, J.-e. Won, D. W. Kim, C. Y. Lee, H. K. Kim, J.-U. Park, Science Advances 2021, 7 (14), eabf7194, https://doi.org/10.1126/sciadv.abf7194.

[6] M.-K. Shin, Y. W. Ji, C.-E. Moon, H. Lee, B. Kang, W.-S. Jinn, J. Ki, B. Mun, M.-H. Kim, H. K. Lee, S. Haam, Biosensors and Bioelectronics 2020, 162, 112254, https://doi.org/https://doi.org/10.1016/j.bios.2020.112254.
